# Novel Evolutionary Lineages Revealed in the Chaetothyriales (Fungi) Based on Multigene Phylogenetic Analyses and Comparison of ITS Secondary Structure

**DOI:** 10.1371/journal.pone.0063547

**Published:** 2013-05-28

**Authors:** Martina Réblová, Wendy A. Untereiner, Kamila Réblová

**Affiliations:** 1 Department of Taxonomy, Institute of Botany, Academy of Sciences, Průhonice, Czech Republic; 2 Department of Biology, Brandon University, Brandon, Manitoba, Canada; 3 Central European Institute of Technology, Masaryk University, Brno, Czech Republic; University of Nebraska, United States of America

## Abstract

*Cyphellophora* and *Phialophora* (Chaetothyriales, Pezizomycota) comprise species known from skin infections of humans and animals and from a variety of environmental sources. These fungi were studied based on the comparison of cultural and morphological features and phylogenetic analyses of five nuclear loci, i.e., internal transcribed spacer rDNA operon (ITS), large and small subunit nuclear ribosomal DNA (nuc28S rDNA, nuc18S rDNA), β-tubulin, DNA replication licensing factor (*mcm7*) and second largest subunit of RNA polymerase II (*rpb2*). Phylogenetic results were supported by comparative analysis of ITS1 and ITS2 secondary structure of representatives of the Chaetothyriales and the identification of substitutions among the taxa analyzed. Base pairs with non-conserved, co-evolving nucleotides that maintain base pairing in the RNA transcript and unique evolutionary motifs in the ITS2 that characterize whole clades or individual taxa were mapped on predicted secondary structure models. Morphological characteristics, structural data and phylogenetic analyses of three datasets, i.e., ITS, ITS-β-tubulin and 28S-18S-*rpb2*-*mcm7*, define a robust clade containing eight species of *Cyphellophora* (including the type) and six species of *Phialophora*. These taxa are now accommodated in the Cyphellophoraceae, a novel evolutionary lineage within the Chaetothyriales. *Cyphellophora* is emended and expanded to encompass species with both septate and nonseptate conidia formed on discrete, intercalary, terminal or lateral phialides. Six new combinations in *Cyphellophora* are proposed and a dichotomous key to species accepted in the genus is provided. *Cyphellophora eugeniae* and *C. hylomeconis*, which grouped in the Chaetothyriaceae, represent another novel lineage and are introduced as the type species of separate genera.

## Introduction

The anamorphic Herpotrichiellaceae (Chaetothyriales) comprise morphologically diverse dematiaceous fungi that include both saprobic (i.e., non-pathogenic) and medically important species. The latter are involved in long-term infections of skin and subcutaneous tissue of humans and animals that include chromatoblastomycoses and phaeohyphomycoses [1], [2], [3], [4], [5]. Members of the order characterized by a lower degree of virulence or suspected to cause infections have been isolated from subcutaneous tissue, nail or skin scrapings [5], [6], [7]. A number of these taxa, which are described in the anamorph genera *Cyphellophora* G.A. de Vries and *Phialophora* Medlar, are of uncertain position within the Chaetothyriales.

*Cyphellophora*, originally introduced for a single species *C. laciniata* G.A. de Vries [6], encompasses dematiaceous fungi with septate, branched hyphae, intercalary, terminal or lateral phialides with indistinct or funnel-shaped collarettes, and septate, hyaline or pale brown conidia ranging in shape from oblong to fusiform or vermiform. Of the twelve members of the genus described to date, five were isolated samples of animal origin (i.e., nails and skin) [4], [6], [8], [9], [10], [11], whereas the other species occur in soil [12], on plants [13], [14], or as plant endophytes [7], [15]. Based on the analysis of small subunit nuclear ribosomal DNA (nuc18S rDNA) sequences, five species of *Cyphellophora* formed a strongly supported monophyletic clade that was sister to representatives of the Herpotrichiellaceae [13]. In a multigene phylogeny [16], *C. laciniata* and *Ceramothyrium carniolicum* (Rehm) Petrak (Chaetothyriaceae) were inferred as sister to the Herpotrichiellaceae within the Chaetothyriales. Analysis of internal transcribed spacer rDNA operon (ITS), β-tubulin and large subunit nuclear ribosomal DNA (nuc28S rDNA) sequences subsequently resolved *Cyphellophora* and *Phialophora* as close relatives within the Chaetothyriales, although both genera were paraphyletic [11], [14], [17], [18].

During investigations of the systematics of *Phialophora* section *Catenulatae* W. Gams we studied the eleven species originally classified in this section [19], a number of which proved to be members of the distantly related lineages Leotiomycetes and Sordariomycetes [20], [21], [22], [23]. In preliminary phylogenies based on sequences of ITS, two ribosomal DNA and three protein-coding genes, only three remaining species were found to be related to the Chaetothyriales (Eurotiomycetes), *viz. Phialophora clavispora* W. Gams, *P. olivacea* W. Gams and *P. oxyspora* W. Gams. The latter two species were members of a monophyletic clade that was sister to the lineage containing *P. verrucosa* Medlar, the type species of *Phialophora* Medlar (Herpotrichiellaceae). The clade that included *P. olivacea* and *P. oxyspora* also encompassed *P. europaea* de Hoog, Mayser & Haase, *P. reptans* de Hoog and *P. sessilis* de Hoog, taxa referred originally to the *P. verrucosa* complex [5], [24], and the recently described *P. ambigua* P. Feng & de Hoog [11]. *Cyphellophora laciniata* and seven other members of the genus were also situated in the same clade.

The six *Phialophora* positioned as sister to the Herpotrichiellaceae are characterized by subhyaline to lightly pigmented, cylindrical-elongate or occasionally flask-shaped phialides with narrowly cylindrical to funnel-shaped or slightly flaring collarettes that can be slightly darker than the lower part of the phialide. Only *P. reptans* and *P. sessilis* possess intercalary phialides with sessile collarettes and phialidic loci borne directly on undifferentiated hyphae. Conidia are subhyaline, obovoidal, clavate, ellipsoidal or fusiform, and adhere in chains or slimy heads. Among these species, only *P. ambigua*, *P. europaea*, *P. oxyspora* and *P. reptans* are involved in superficial infections of humans [5], [11], [24]. The three other *Phialophora* in this clade have been isolated from plants, fungi, moist wallpaper or nutrient-poor substrates such as marble, stalactites or resin [5], [19], [24], [25]. All of these taxa can be differentiated from *P. verrucosa* and *P. americana* (Nannf.) S. Hughes (Herpotrichiellaceae) by their slow-growing colonies and phialides with smaller, funnel-shaped and less deeply pigmented collarettes [5], [18], [24], [25]. They resemble species of *Cyphellophora* in possessing similar conidiogenous cells with smaller, slightly darker collarettes, but differ mainly by their shorter, nonseptate conidia.

The study described in this paper was undertaken to clarify the phylogenetic relationships of *Cyphellophora* and several phialophora-like species that have been resolved repeatedly as comprising a group that is sister to the Herpotrichiellaceae. We investigated relationships among the clades within the Chaetothyriales based on the combination of several approaches including the comparison of morphological and cultural characteristics and phylogenetic analyses of sequences of the ITS, nuc18S rDNA, nuc28S rDNA and three protein-coding genes, i.e., β-tubulin, DNA replication licensing factor (*mcm7*) and second largest subunit of RNA polymerase II (*rpb2*). We also performed in-depth comparative analyses of ITS1 and ITS2 secondary (2D) structures of members of this order. ITS1 and ITS2 represent rapidly evolving regions of the rDNA operon and display high sequence variability. Although these loci are non-coding, they include functionally constrained positions required for the folding of their transcripts that allow their own splicing and the correct processing of the rDNA genes [26]. The ITS2 has been shown to retain a common core structure with hallmarks in its RNA transcripts that are evolutionarily constrained and universal among eukaryotes [27], [28], [29]. These include four helices (H1–H4), a pyrimidine–pyrimidine mismatch in helix H2 and the occurrence of the YGGUY motif in helix H3. At the structural level, these characters have proven useful for molecular taxonomic concepts [30]. Evolutionary processes at the RNA structural level, that are responsible for preserving the RNA helix structure, i.e., the double-sided (compensatory base change, CBC) and one-sided substitution (hemi-compensatory base change, hCBC), have been accepted as a basis for the CBC species concept [30], [31]. This concept, which has been used to delimit biological species, is based on co-evolution of nucleotides involved in CBCs and hCBCs in two most conserved helices of the ITS2 molecule [30] and was used in our study to examine relationships among closely related *Cyphellophora*.

Finally, to study relationships among major clades of the order and among species of *Cyphellophora* and *Phialophora* at the structural level of the rRNA, we (1) built consensus 2D structures of ITS1 and ITS2, (2) searched for non-conserved co-evolving nucleotides that maintain base pairing in the RNA transcript, and (3) mapped all existing substitutions on to the predicted 2D models of ITS1 and ITS2.

## Materials and Methods

### Morphological characterization of fungal strains

Conidiophores, conidiogenous cells and conidia were examined in water, Melzer's reagent or 90% lactic acid. Images were captured using differential interference (DIC) or phase contrast (PC) microscopy using an Olympus DP70 Camera operated by Imaging Software Cell on an Olympus BX51 compound microscope.

Strains were grown on potato-carrot agar (PCA), oatmeal agar (OA) and 2% malt extract agar (MEA) [32] in the dark and under near-UV light source (12 h light: 12 h dark). Colonies were examined after 7, 21 and 30 d at 25°C. Images of taxa presented in this study are of 30 d old cultures on PCA, if not stated otherwise. Cultures are maintained at the Centre for Agricultural Bioscience International (CABI, formerly IMI), Egham, Surrey, UK, CBS-KNAW Fungal Biodiversity Center (CBS), Utrecht, The Netherlands, the culture collection of P.W. Crous (CPC at CBS), the Canadian Collection of Fungal Cultures (DAOM), Agriculture and Agri-Food Canada, Ottawa, Canada, the Mae Fah Luang University Culture Collection, Thailand (MFLUCC), Mycothèque de l′Université catholique de Louvain (MUCL), Louvain-la-Neuve, Belgium, the University of Alberta Microfungus Collection and Herbarium (UAMH), Edmonton, Canada, and the culture collection of W.A. Untereiner (WUC) at Brandon University, Canada.

### DNA extraction, amplification and sequencing

Cultures used for DNA isolations were grown as described previously [23], [33] and total nucleic acids were extracted from mycelia following the protocols of Lee and Taylor [34] or Réblová *et al*. [23]. Procedures for amplifying and sequencing the ITS, nuc18S, nuc28S and *rpb2* were performed as described in Bogale *et al*. [22] and Réblová *et al*. [23]. The *mcm7* was amplified and sequenced using primers Mcm7-709for and Mcm7-1348rev/Mcm7-1447rev [35]. Sequences were edited using Sequencher 5.0 software (Gene Codes Corp., Ann Arbor, MI, USA).

### Sequence alignment

ITS, nuc28S, nuc18S, β-tubulin, *mcm7* and *rpb2* sequences determined for this study and homologous sequences retrieved from GeneBank are listed in Table S1. The selection of retrieved sequences was based on top hits for each gene obtained from BLAST searches. The retrieved sequences were mostly published in studies by Hoog *et al*. [5], [24], Decock *et al*. [13], Crous *et al*. [14], [17], [36], Untereiner *et al*. [18], Tsuneda *et al*. [37] and Chomnunti *et al*. [38], [39].

Sequences were manually aligned in BioEdit v.7.0.9.0 [40]. Nuc18S and nuc28S alignments were enhanced by utilizing the homologous 2D structure of *Saccharomyces cerevisiae* Meyen ex E.C. Hansen [41], [42] to improve decisions regarding homologous characters and introduction of gaps. 2D models obtained for the ITS1 and ITS2 (see below) were used to determine the positions of homologous nucleotides in the ITS. The alignment of sequences of β-tubulin, *rpb2* and *mcm7* genes were obtained as described in Réblová and Réblová [43].

Three multiple sequence alignments (MSAs) were constructed: 1) ITS, 2) ITS combined with β-tubulin, and 3) nuc18S and nuc28S combined with *mcm7* and *rpb2*. Individual alignments were concatenated to combine sequences for the bi- and multilocus alignments. Alignments are deposited in TreeBASE (Study no. 13812).

### Phylogenetic analyses

Phylogenetic relationships were resolved based on analyses of ITS, β-tubulin, nuc18S, nuc28S, *mcm7* and *rpb2* sequences of 70 isolates representing 46 species from three families of the Chaetothyriales. We analyzed the first two-thirds of the 5′ half of the nuc28S (corresponding to the first 1197 nucleotides of *Saccharomyces cerevisiae*), the almost entire nuc18S, partial *mcm7* and the 5−7 segments of the *rpb2*. Bases 1−76 of the nuc18S and nuc28S alignments, 1−60 of the *rpb2* and 1–78 of the β-tubulin alignments were excluded from analyses because of the incompleteness of the 5′-end of the majority of the available sequences. Two outgroups were used for the three phylogenies. Members of the Herpotrichiellaceae were used to root the ITS and ITS-β-tubulin phylogenetic trees. *Saccharomyces cerevisiae* (nuc18S J01353, nuc28S J01355, *rpb2* M15693) and *Vanderwaltozyma polyspora* (Van der Walt) Kurtzman (nuc18S X83825, nuc28S AY048169, *rpb2* EF599484) (Saccharomycetes) were used to root the multilocus phylogeny.

Each combined data set was partitioned into several subsets of nucleotide sites. The ITS-β-tubulin analysis was partitioned into 1) ITS, and 2−4) first, second and third codon positions of β-tubulin. The four-gene data set was partitioned into 1) nuc28S, 2) nuc18S, 3–5) first, second and third codon positions of *rpb2*, and 6–8) first, second and third codon positions of *mcm7*.

MrModeltest2 v.2.3 [44] was used to infer the appropriate substitution model that would best fit the model of DNA evolution for each sequence data set and each partition of the combined data sets. Maximum likelihood (ML) and Bayesian inference (BI) analyses were used to estimate phylogenetic relationships. ML analysis was performed with RAxML-HPC v.7.0.3 [45], [46] with a Gtrcat model of evolution. Nodal support was determined by nonparametric bootstrapping (BS) with 1 000 replicates. BI analysis was performed in a likelihood framework as implemented in MrBayes v.3.0b4 to reconstruct phylogenetic trees [47]. For the ITS data set we selected the Gtr+g substitution model for BI analysis. For the ITS-β-tubulin data set we used a Gtr+g substitution model for the first codon position of β-tubulin, a Hky+g model for the second position and the Hky+i+g model for the third. For the combined nuc18S, nuc28S, *mcm7* and *rpb2* data set we used different models for each partition: Gtr+i+g for nuc28S, nuc18S and first codon position of *mcm7*; Gtr+g for the first and second codon position of *rpb2* and second codon of *mcm7*; Hky+i+g for the third codon position of *rpb2;* Gtr+i for the third codon position of *mcm7*. Multiple Bayesian searches using Metropolis-coupled Markov chain Monte Carlo sampling were conducted. One cold and three heated Markov chains were used in the analysis. Analyses were run for 5 million generations, with trees sampled every 1 000 generations. The first 20 000 trees, which represented the burn-in phase of the analysis, were discarded. The remaining trees were used for calculating posterior probabilities (PP) of recovered branches [48] in the 50% majority rule consensus tree.

### Prediction of 2D structure models of ITS1 and ITS2

Predicting the 2D structure of the ITS is essential for constructing a reliable MSA to compare nucleotides at homologous positions (in helices and loops) while searching for non-conserved co-evolving nucleotides that maintain base pairing. Consensus 2D structure models for the ITS1 and ITS2 were built using the PPfold program v.3.0 [49] which uses an explicit evolutionary model and a probabilistic model of structures, and relies on multiple sequence alignment of related RNA sequences. Final 2D models created for all members of the Chaetothyriales were further improved using Mfold program [50] and then adjusted manually if necessary, based on comparison of homologous positions in the MSA. The predicted 2D RNA structures were obtained in a dot bracket notation and were visualized and drawn using VARNA: Visualization Applet for RNA program [51]. The ITS2 Database III [52] with available structural models was also used to build consensus 2D structures for members of the Chaetothyriales.

### Identification and mapping of base-pair changes in 2D structures of ITS

We identified three types of substitutions in the aligned ITS sequences. The first involves compensatory base changes (CBCs) that occur when both nucleotides of a paired site mutate (i.e., G = C ↔ C = G, A-U or U-A). Because these canonical base pairs are isosterical [53] they can be substituted for each other within stems without causing structural perturbations. The second type of substitution, the hemi-compensatory base change (hCBC), entails the change of a canonical base pair to a ‘wobble’ base pair (i.e., G = C → G/U). This type of substitution, which is classified as near-isosteric [54], retains base pairing in RNA molecules but produces minor structural perturbations in the helix structure because the canonical G = C pair and wobble G/U pair are not members of the same isosterical family [53]. Finally, we identified non-compensatory base changes (non-CBCs) that involve the replacement of a canonical pair or a wobble pair with any non-canonical pair. CBCs and hCBCs are responsible for maintaining the arrangement of base pairs in the RNA transcript and preserving the structure of the RNA helices whereas non-CBCs lead to the disruption of the stem structure [53]. Once all existing substitutions were identified among members of the Chaetothyriales, those unique to the Herpotrichiellaceae, the newly proposed family and phylogenetically related taxa of the Chaetothyriaceae s. str. were mapped onto the predicted 2D structures of ITS1 and ITS2 of *Phialophora verrucosa* and *Cyphellophora laciniata.*

### Nomenclature

The electronic version of this article in Portable Document Format (PDF) in a work with an ISSN or ISBN will represent a published work according to the International Code of Nomenclature for algae, fungi, and plants, and hence the new names contained in the electronic publication of a *PLOS ONE* article are effectively published under that Code from the electronic edition alone, so there is no longer any need to provide printed copies.

In addition, new names contained in this work have been submitted to MycoBank from where they will be made available to the Global Names Index. The unique MycoBank number can be resolved and the associated information viewed through any standard web browser by appending the MycoBank number contained in this publication to the prefix http://www.mycobank.org/MycoTaxo.aspx?Link=T&Rec=. The online version of this work is archived and available from the following digital repositories: PubMed Central, LOCKSS.

## Results

### Phylogenetic results

**Figure 1:**
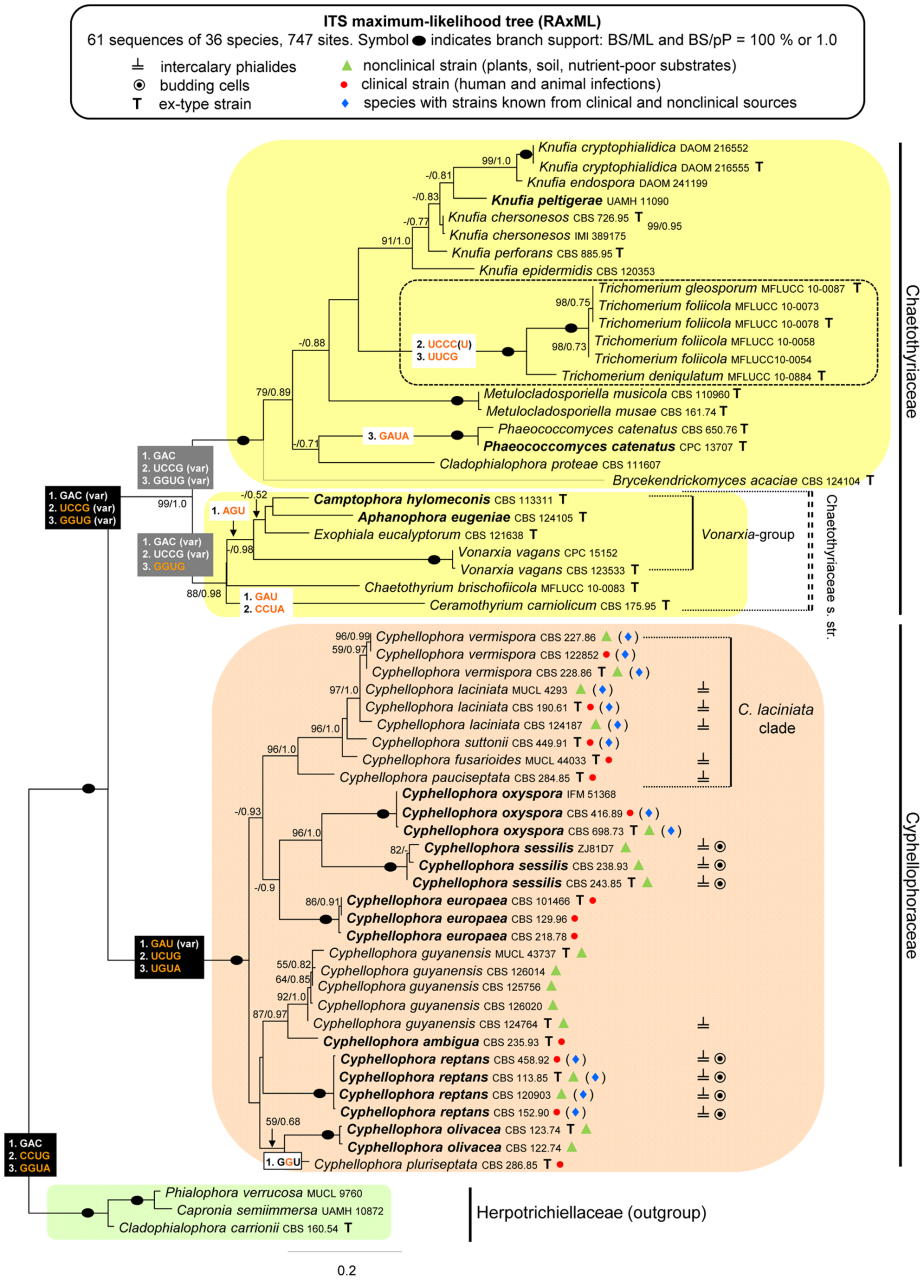
Phylogenetic analysis of ITS sequences of 36 members of the Chaetothyriales. Phylogram inferred from the ML analysis with RAxML using the Gtrcat model of evolution. Taxa in bold refer to new combinations or new taxonomic treatments. The three evolutionary motifs identified in the 2D structure of ITS2 and unique for certain phylogenetic groups are mapped on the phylogram. The abbreviation (var) indicates certain motifs deviations.

The ITS data set for 36 species consisted of 61 sequences each with 747 characters. The ML tree is shown in Fig. 1. Three major groups were resolved: the Herpotrichiellaceae (ML BS 100%/PP 1.0), a well-supported lineage (99/1.0) corresponding to the Chaetothyriaceae, and a robust clade (100/1.0) comprising *Cyphellophora laciniata*, the type species of the genus, with six other *Cyphellophora* and six phialophora-like taxa. This latter group represents a new family that we introduce as the Cyphellophoraceae. The core of this family is the *C. laciniata* subclade (96/1.0) that contains *C. fusarioides* (B. Sutton & C.K. Campb.) Decock, *C. laciniata*, *C. suttonii* (Ajello, A.A. Padhye & M. Payne) Decock, *C. pauciseptata* P. Feng & de Hoog and *C. vermispora* A. Walz & de Hoog. The Chaetothyriaceae encompass two subclades. The first (88/0.98) contains the *Vonarxia*-group (−/0.98) including *Exophiala eucalyptorum* Crous, *Vonarxia vagans* (Speg.) Aa and two *Cyphellophora* species, *C. eugeniae* Crous & Alfenas and *C. hylomeconis* Crous, de Hoog & H.D. Shin. It is sister to *Ceramothyrium carniolicum* (Rehm) Petr. and *Chaetothyrium brischofiicola* (as *brischofiacola*) Chomnunti & K.D. Hyde [39]. The second subclade (100/1.0) encompasses species of *Cladophialophora* Borelli, *Knufia* L.J. Hutchison & Unter., *Phaeococcomyces* de Hoog, *Metulocladosporiella* Crous, Schroers, Groenewald, U. Braun & K. Schubert and *Trichomerium* Speg. This lineage also contains *Capronia peltigerae* (Fuckel) D. Hawksw., which grouped with species of *Knufia* (91/1.0).

**Figure 2:**
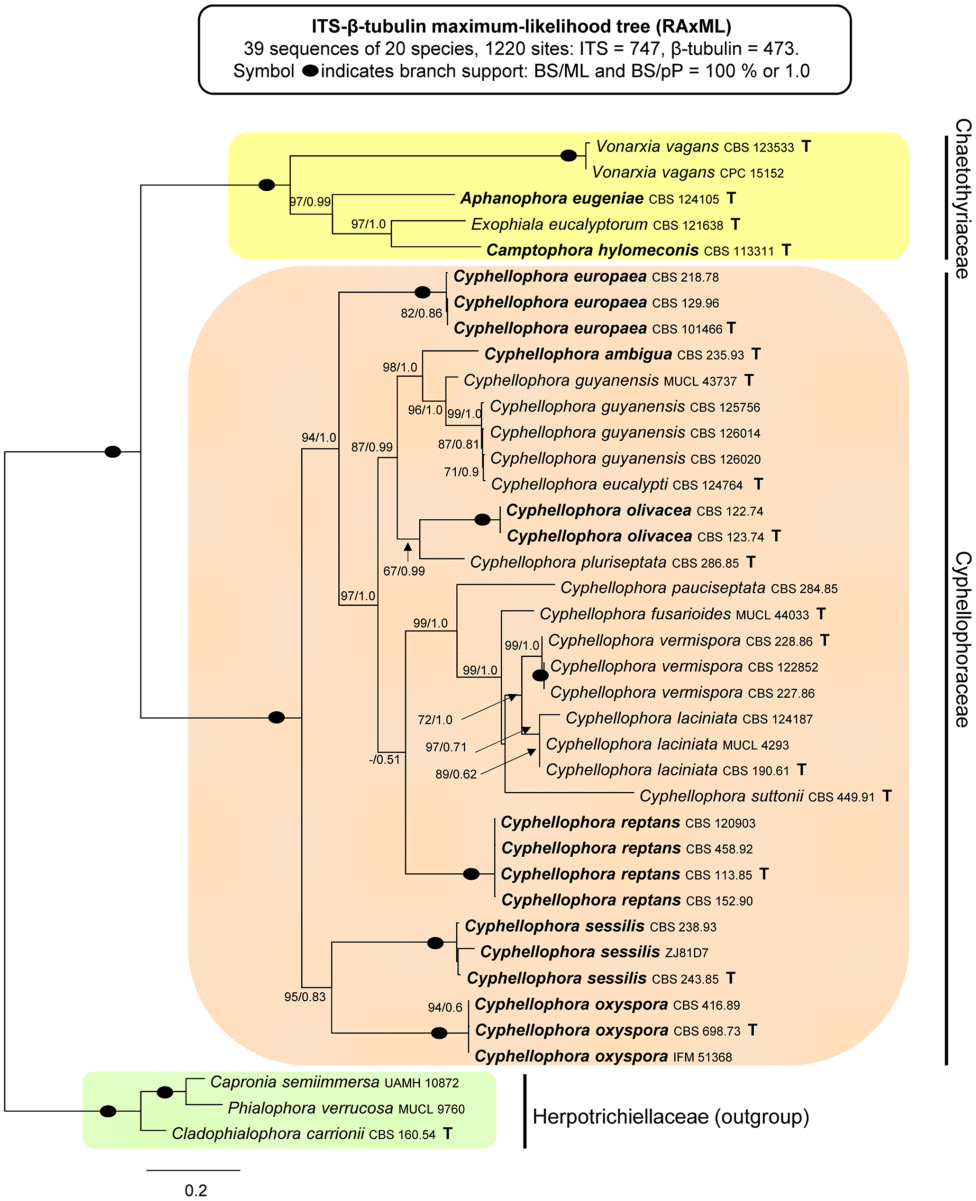
Phylogenetic analysis of the combined ITS rDNA and β-tubulin sequences of 20 members of the Chaetothyriales. Phylogram inferred from the ML analysis with RAxML using the Gtrcat model of evolution. Taxa in bold refer to new combinations or new taxonomic treatments.

The alignment for the combined ITS and β-tubulin data set consisted of 39 sequences representing 20 members of the Chaetothyriales and 1220 characters. In the ML tree shown in Fig. 2, the Cyphellophoraceae, Herpotrichiellaceae and Chaetothyriaceae are resolved as robust clades (100/1.0). The groupings of taxa within the Cyphellophoraceae are similar to those inferred in the ITS analysis, but supports for branches in the combined ITS-β-tubulin phylogeny are generally stronger. Both *C. eugeniae* and *C. hylomeconis* are positioned again outside the Cyphellophoraceae in a clade containing members of the Chaetothyriaceae.

**Figure 3:**
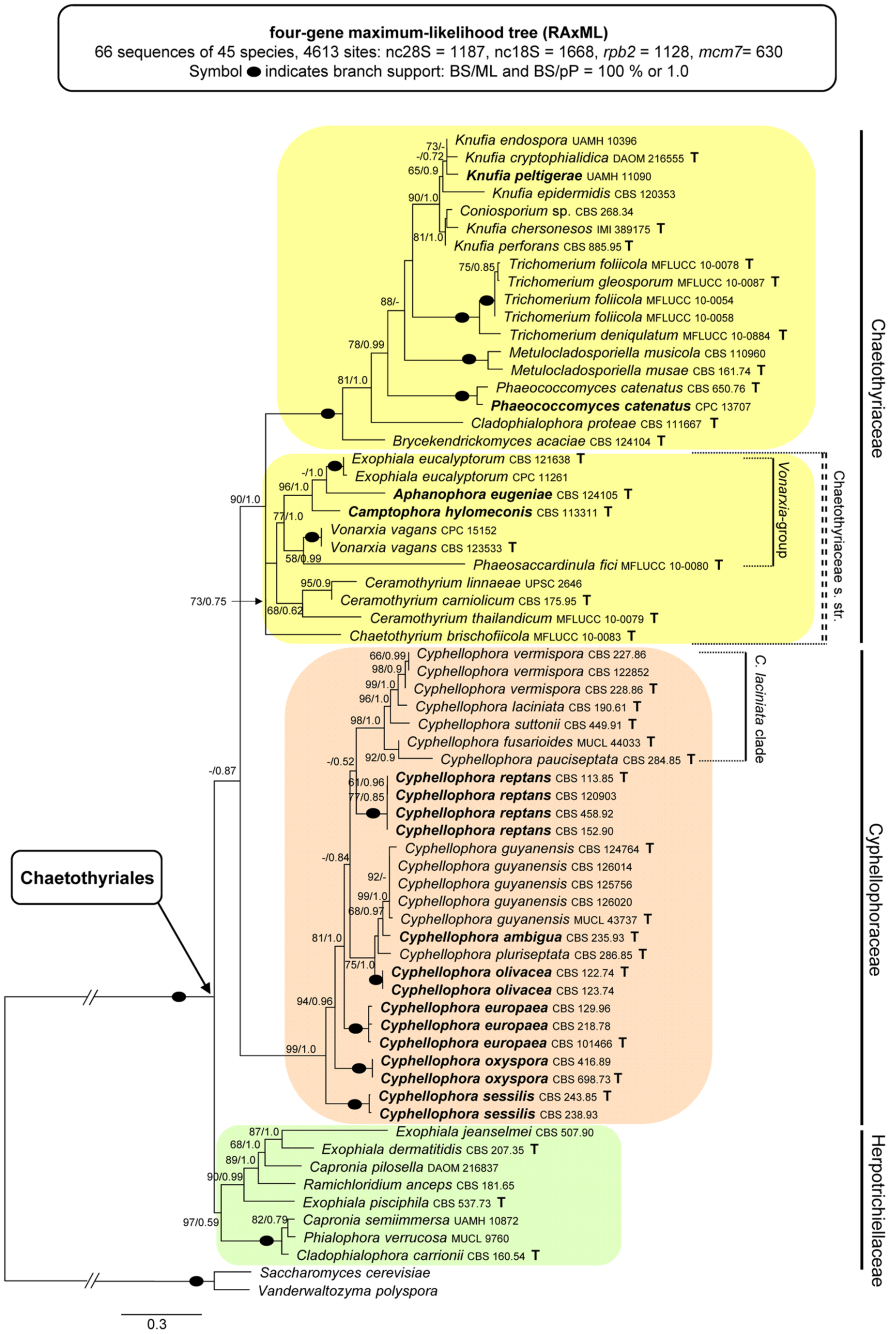
Multilocus phylogenetic analysis of the nuc28S-nuc18S-*rpb2*-*mcm7* sequences of 45 members of the Chaetothyriales. Phylogram inferred from the ML analysis with RAxML using the Gtrcat model of evolution. Taxa in bold refer to new combinations or new taxonomic treatments.

In the third analysis, the combined nuc18S, nuc28S, *mcm7* and *rpb2* sequences were assessed for 66 taxa representing 45 species in the Chaetothyriales. This multiple alignment consisted of 4613 characters. The ML tree is shown in Fig. 3. In this phylogeny the Chaetothyriales is a strongly supported monophyletic clade (100/1.0) comprising three major subclades; the Cyphellophoraceae (99/1.0), Herpotrichiellaceae (97/0.59) and a large clade (90/1.0) representing the Chaetothyriaceae. As in the ITS phylogeny, the Chaetothyriaceae is divided into two lineages. The first (73/0.75) contains three species of *Ceramothyrium* Bat. & H. Maia (68/0.62), the *Vonarxia*-group (77/1.0), *Phaeosaccardinula fici* (as *ficus*) Chomnunti & K.D. Hyde [38] and *Chaetothyrium brischofiicola*. The second subclade (100/1.0) includes the holomorph genera *Knufia* and *Trichomerium* and four genera of dematiaceous hyphomycetes. A strain of *Glyphium elatum* (Grev.) H. Zogg (CBS 268.34) was positioned within the genus *Knufia*.

### Consensus 2D structure of ITS1

**Figure 4:**
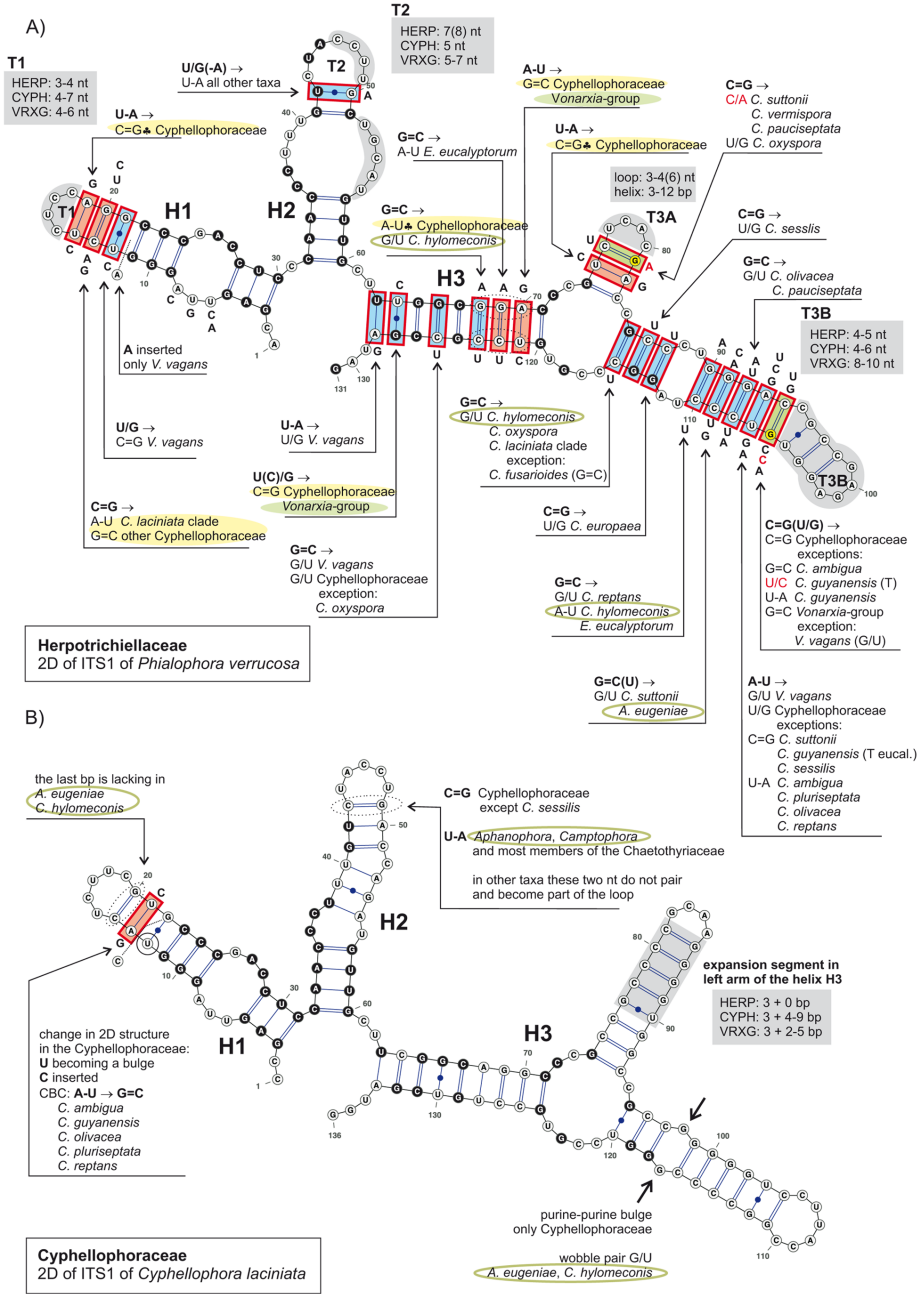
Partial secondary structure of ITS1 rRNA molecule showing three helices (H1–H3). A) Partial 2D structure of *Phialophora verrucosa*. All substitutions recorded among representatives of the Cyphellophoraceae, Herpotrichiellaceae and members of the *Vonarxia*-group are mapped on the 2D model. B) Partial 2D structure of *Cyphellophora laciniata*. Variability in the 2D structure among members of the *Cyphellophoraceae* and two other taxa, *A. eugeniae* and *C. hylomeconis*, segregated from *Cyphellophora*, is recorded on the 2D model. Abbreviations: HERP  =  Herpotrichiellaceae, CYPH  =  Cyphellophoraceae, VRXG  =  *Vonarxia*-group. Parts of hairpin loops highlighted with gray color represent regions with variable number of nucleotides. The legend to symbols and colors is included in Figure 6.

**Figure 5:**
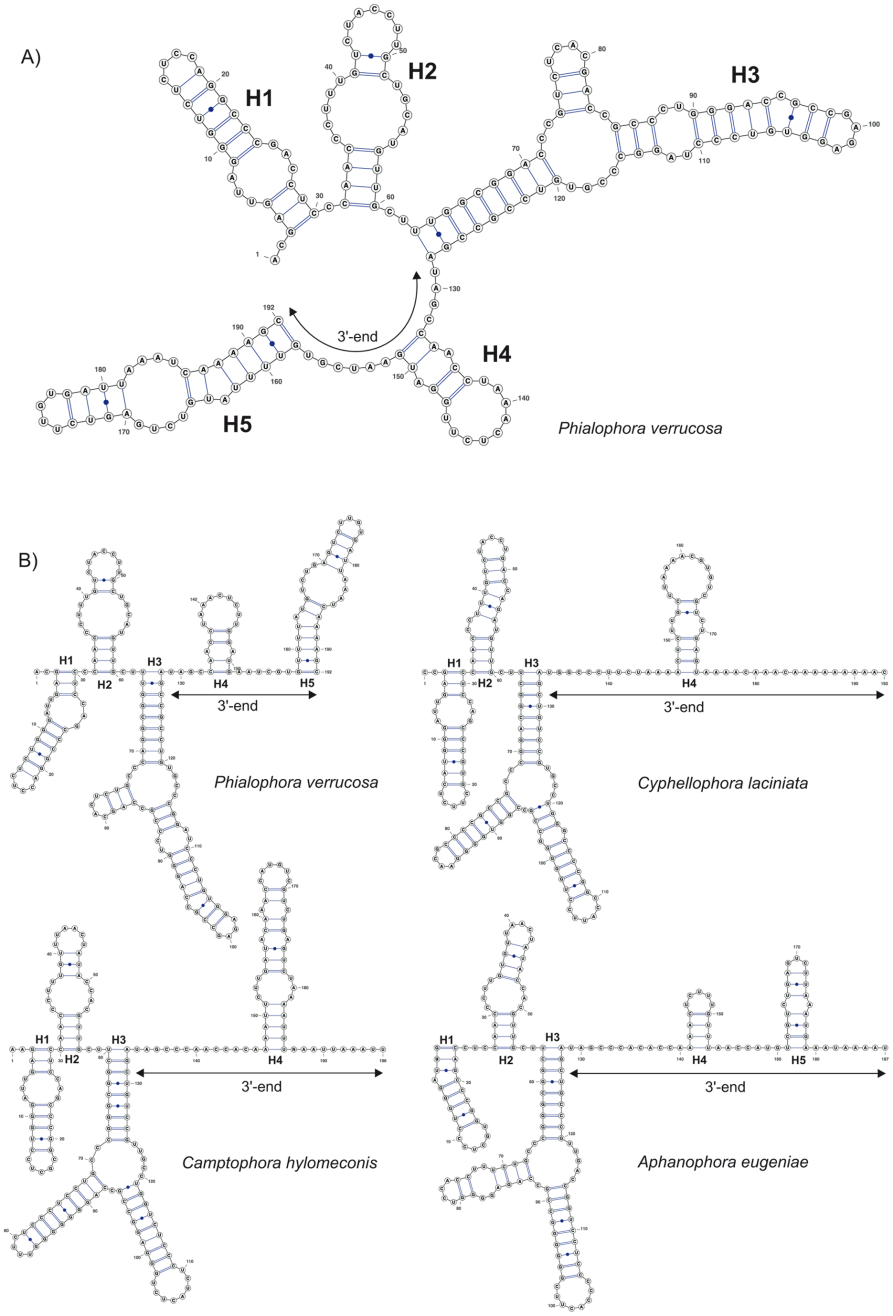
Predicted secondary structure models of ITS1 rRNA. A) The predicted ‘ring’ model of *Phialophora verrucosa*, the variable 3′-end is indicated. B) The predicted ‘ring’ models of 2D structure of ITS1 of representative species of the Cyphellophoraceae (*C. laciniata*), Herpotrichiellaceae (*P. verrucosa*), and *Aphanophora* and *Camptophora* (*Vonarxia*-group), are transformed into linear models with helices.

The 2D predicted structures of ITS1 shown in Fig. 4 are modeled for *Cyphellophora laciniata* and *Phialophora verrucosa,* representatives of the Cyphellophoraceae and Herpotrichiellaceae, respectively. The consensus 2D structure of ITS1 consisted of three helices and a variable single-stranded segment at 3′-end (Fig. 5A). The juxtaposed 3′-end folded into one or two helices that were variably positioned and separated by single-stranded regions of variable length. Because these helices are generated from non-homologous regions they cannot be compared among taxa (Fig. 5B).

The consensus 2D model of ITS1 mapped onto *P. verrucosa* (Fig. 4A) contains three helices (H1, H2 and H3) separated by an adjacent single-stranded (junction) region. H1 is *ca* 30-nucleotides (nt) long consisting of nine base pairs (bp). It exhibits a symmetrical internal loop and a hairpin loop T1 of a variable length. H2 is also *ca* 30-nt long but it consists of six bp. It includes an internal loop and a hairpin loop T2 that are variable in length. H3 is the longest (*ca* 70 nt long) and most variable of the helices; it is also the only branched duplex in the ITS1. In the Herpotrichiellaceae it consists of a three-way junction and a short symmetrical (2×2) internal loop in the right arm. The latter changes to a purine-purine pair in all Cyphellophoraceae. The length of the left arm of H3 is shortest in the Herpotrichiellaceae (3 bp) but a large insertion beyond these three bp occurs in the Cyphellophoraceae and Chaetothyriaceae (Fig. 4B). The two hairpin loops T3A and T3B in H3 exhibit sequence variation. H1 and H2 contain six and five conserved bp in all Chaetothyriales, respectively. Other pairs in these helices are either CBCs or hCBCs. In contrast, H3 has only three conserved bp; the two pairs in this helix are non-CBCs, others show CBCs/hCBCs.

### Consensus 2D structure of ITS2

**Figure 6:**
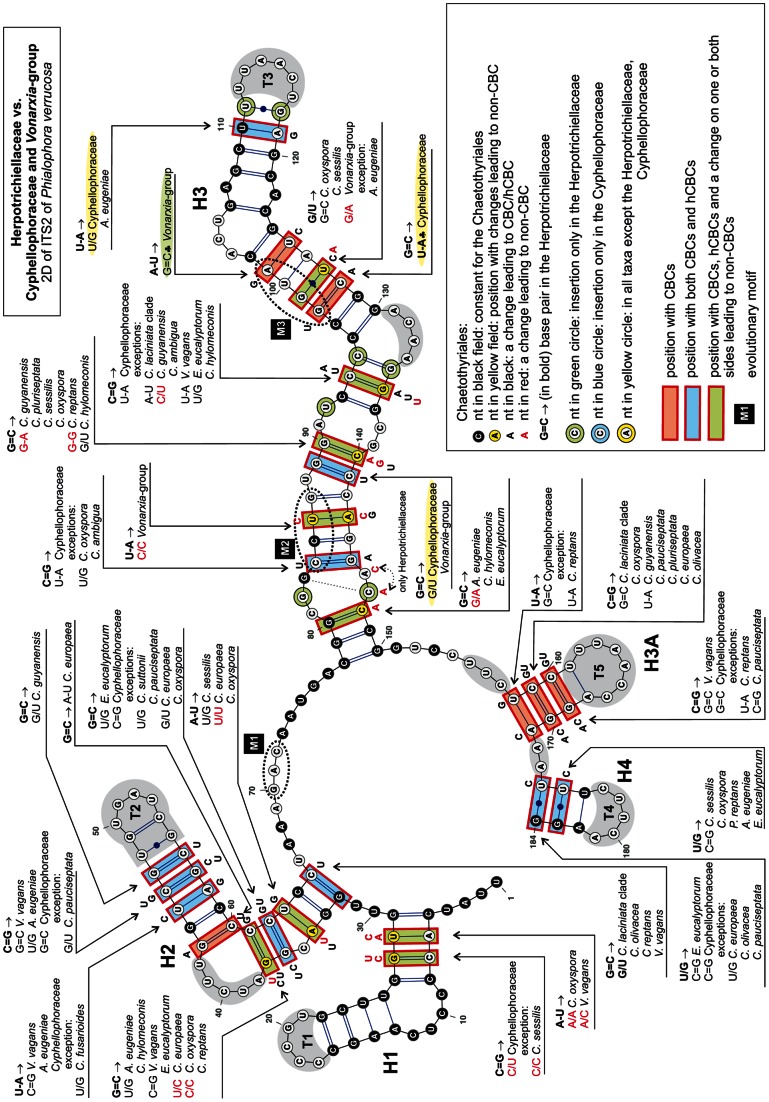
Predicted secondary structure model of the ITS2 rRNA molecule of *Phialophora verrucosa*. Five helices commonly found in the 2D structure in members of the Cyphellophoraceae and Herpotrichiellaceae are numbered H1–H3, H3A, H4. All substitutions recorded among representatives of these two families and members of the *Vonarxia*-group are mapped on the 2D model. Parts of hairpin loops highlighted with gray color represent regions with variable number of nucleotides.

**Figure 7:**
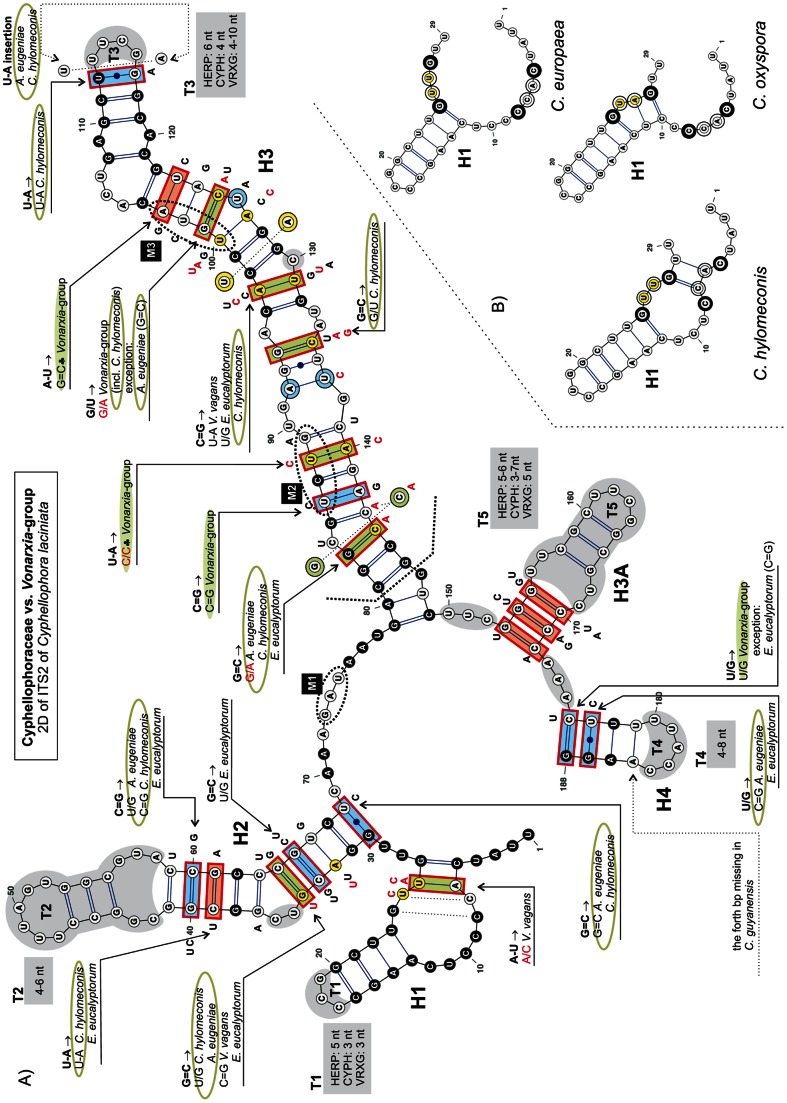
Predicted secondary structure model of the ITS2 rRNA molecule of *Cyphellophora laciniata*. Substitutions that characterize Cyphellophoraceae and members of the *Vonarxia*-group are mapped on the 2D model. Parts of hairpin loops highlighted with gray color represent regions with variable number of nucleotides. The legend to symbols and colors is included in Figure 6.

The consensus secondary structure of ITS2 molecule mapped onto *P. verrucosa* (Fig. 6) and *C. laciniata* (Fig. 7) folded into a ring structure with four main helices (H1−H4) and one alternative helix (H3A) that is inserted between H3 and H4 and separated by an adjacent single-stranded region. The ITS2 Database III [52] with available structural models was also used to build consensus 2D structures for members of the Chaetothyriales. However, the predicted structures resulted in conflicting folding patterns for individual species. In general, the 2D structures folded into four to five helices, which were generated from non-homologous sequence regions. Because such structures cannot be compared across taxa we did not use these models in our study.

H1 is *ca* 25-nt long with 8 bp, and consists of a conserved asymmetrical internal loop and a hairpin loop T1, which is variable. Although the primary sequence of H1 is constant in length among members of the Chaetothyriales, the 2D structure of the bottom portion of H1 is highly variable among members of the Cyphellophoraceae and the *Vonarxia*-group (i.e., two middle base pairs show non-CBCs that result in the disruption of the helix as illustrated for *Cyphellophora europaea*, *C. oxyspora* and *Camptophora hylomeconis*) (Fig. 7B).

H2 consists of *ca* 21–37 nt. It is composed of an asymmetrical loop and a hairpin loop T2, both of which exhibit a high degree of sequence variation, and a ‘C’ bulge on the 5′ side that is observed only in the Herpotrichiellaceae. The asymmetrical loop on the 5′ side consists of 4–5 nt in the Herpotrichiellaceae and 1–3 nt in other Chaetothyriales. Within the *C. laciniata* clade and in *C. pauciseptata*, this loop contains two nt, whereas other Cyphellophoraceae possess only one nt that forms a bulge. Apart from conservative bp (five in H1 and two in H2), both H1 and H2 contain two non-CBCs. In addition, the H2 helix contains several hCBCs and one CBC.

H3 is the longest duplex. It comprises *ca* 73 nt and consists of up to five internal loops, one bulge and a hairpin loop T3. The shortest internal loop (1×1) is formed by a conserved A/A pair. Two internal loops are asymmetrical and two others are variable in sequences among the organisms compared. In general, it is difficult to say whether they are symmetrical or asymmetrical. This helix contains nine conserved bp, two bp with CBCs, and three bp with both CBCs and hCBCs. Other pairs are non-CBCs. The most conserved area in the ITS2 is located near the 5′-end of H3. Nucleotides that are constant in all members of the Chaetothyriales are found on both sides of the upper part of H3. The fourth duplex H4 is short having one conserved bp and two bp with both CBCs and hCBCs. Its hairpin loop T4 is of variable length.

The alternative duplex H3A is positioned between H3 and H4. It is present only in the Herpotrichiellaceae, Cyphellophoraceae, and selected Chaetothyriaceae, i.e., *V. vagans, Ceramothyrium thailandicum* Chomnunti & K.D. Hyde and *Phaeococcomyces catenatus* (de Hoog & Herm.-Nijh.) de Hoog.

The 2D model of *Brycekendrickomyces acaciae* Crous & M.J. Wingf. differs from those produced for the Herpotrichiellaceae, Chaetothyriaceae and Cyphellophoraceae. In this species the H3A is short and the remaining nucleotides form an adjacent single-stranded region and do not fold into H4. Two species of *Metulocladosporiella* possess the longest H4 helix and the adjacent single-stranded region following H3 is missing in these taxa. All nucleotides past the conserved GU(C)C triplet (nt 151−153 of *Phialophora verrucosa*, Fig. 6) form a 12 bp long helix H4 consisting of one symmetrical and one asymmetrical internal loop.

We identified three evolutionary motifs in the 2D structure of ITS2 that are unique for certain phylogenetic groups (Table 1 and Figs. 1, 6, 7). Motif M1 (GAC, with a deviation in *Ceramothyrium carniolicum* as GAU) is repeated in all members of the Chaetothyriales with exception of the *Vonarxia*-group (AGU), and the Cyphellophoraceae (GA(G)U), and is positioned in a single-stranded region between H2 and H3. Motifs M2 and M3 occur on the 5′side of H3 (Figs. 6, 7). M2 is positioned in the lower half of the H3; this motif is unique for the Cyphellophoraceae (UCUG) and the Herpotrichiellaceae (CCUG), but varies within the Chaetothyriaceae (UCCG), i.e., CCUA in *Ceramothyrium carniolicum*, UCAG in *Cladophialophora proteae* Viljoen & Crous and UCCC(U) in *Trichomerium*. M3 is situated in the lower portion of the highly conserved region near the hairpin loop T3 and defines the Cyphellophoraceae (UGUA), Herpotrichiellaceae (GGUA) and some Chaetothyriaceae (GGUG).

**Table 1:** A list of evolutionary motifs (M1–M3) recorded in the ITS2 molecule of members of the Chaetothyriales.

Taxa and families	M1	M2	M3
Herpotrichiellaceae	GAC	**CCUG**	**GGUA**
Cyphellophoraceae	GAU^a^	**UCUG**	**UGUA**
Chaetothyriaceae	GAC*	**UCCG** *	**GGUG** *
* Ceramothyrium carniolicum*	GAU*	CCUA*	GGUG
* Cladophialophora proteae*	GAC	UCAG*	GGUG
* Phaeococcomyces catenatus*	GAC	UCCG	GAUA*
* Trichomerium*	GAC	UCCC^b^ *	UUCG*
* Vonarxia*-group	AGU*	UCCG	GGUG

Motifs in bold determine a group at family level. Taxa or groups of taxa that belong to the Chaetothyriaceae and show certain motifs deviations are listed.

* Asterisk indicates presence of more than one unique motif within the group. ^(a,b)^ deviations: ^a^GGU in *Cyphellophora pluriseptata*, ^b^UCCU in *Trichomerium deniqulatum.*

### Taxonomy

#### Cyphellophoraceae

Réblová & Unter., fam. nov.

[urn:lsid:indexfungorum.org:names: 803682]

*Mycelium* composed of hyaline or pigmented hyphae. *Conidiophores* mostly formed as simple phialides. *Phialides* discrete, intercalary, terminal or lateral, sometimes very short, flask-shaped, elongate flask-shaped, or cylindrical elongate and then hardly tapering towards the collarette. *Collarettes* funnel-shaped with flaring opening or cylindrical and hardly divergent, hyaline or slightly darker than the lower part of the phialide. *Conidia* nonseptate or septate, hyaline, subhyaline or pale brown, ranging from obovoidal or ellipsoidal to fusiform, cylindrical, acicular, straight, curved or sigmoid. Budding and germinating cells present. Teleomorphs unknown.

Type genus. *Cyphellophora* G.A. de Vries emend. Réblová & Unter.

##### Cyphellophora

G.A. de Vries emend. Réblová & Unter.

[urn:lsid:indexfungorum.org:names:7885]

 =  *Kumbhamaya* M. Jacob & D. J. Bhat, Cryptog. Mycol. 21: 82. 2000.

*Mycelium* composed of branched, septate, hyaline or slightly pigmented hyphae. *Conidiophores* mostly formed as simple phialides, rarely with an additional septum. *Phialides* discrete, intercalary, terminal or lateral, sometimes very short, flask-shaped, elongate flask-shaped, or cylindrical elongate, hardly tapering towards the collarette. *Collarettes* indistinct, funnel-shaped and flaring at the opening or narrowly funnel-shaped to cylindrical and hardly divergent, hyaline or slightly darker than the lower part of the phialide. *Conidia* nonseptate or septate, hyaline, subhyaline or pale brown, ranging from obovoidal to ellipsoidal, clavate, oblong-ovoid, fusiform, cylindrical, vermiform or acicular, straight, curved, falcate or sigmoid, frequently anastomosing. Budding and germinating cells present. Teleomorphs unknown.

Type species. *Cyphellophora laciniata* G.A. de Vries, Mycopath. Mycol. appl. 16: 47. 1962.

Comments. The monophyletic and strongly supported clade encompassing *C. laciniata* and eight other members of the genus also includes six species formerly treated as members of the genus *Phialophora* (Figs. 1–3). Subclades corresponding to individual species are not defined by ecology or by similarities in the morphologies of the conidia (septation, shape, presence of budding cells), collarettes (pigmentation, shape), and conidiogenous cells (shape, intercalary with sessile collarettes *vs.* prominent terminal and/or lateral). Because these subclades are ecologically and morphologically heterogeneous and cannot be recognized as separate genera, we accept all members of the Cyphellophoraceae clade as belonging to *Cyphellophora* and emend the genus to include species with nonseptate conidia and mostly cylindrical-elongate to flask-shaped elongate phialides. Accordingly, we propose six new combinations in *Cyphellophora* and provide a key to its species.

##### Cyphellophora ambigua

(P. Feng & de Hoog) Réblová & Unter., comb. nov.

[urn:lsid:indexfungorum.org:names: 803697]

Basionym. *Phialophora ambigua* P. Feng & de Hoog, Fung. Diver. (p. 22 of the still unpaginated paper), 2012. DOI 10.1007/s13225-012-0194-5.

Habitat. Isolated from a human toenail.

Illustrations. The species is illustrated in Feng *et al*. [11].

##### Cyphellophora europaea

**Figure 8:**
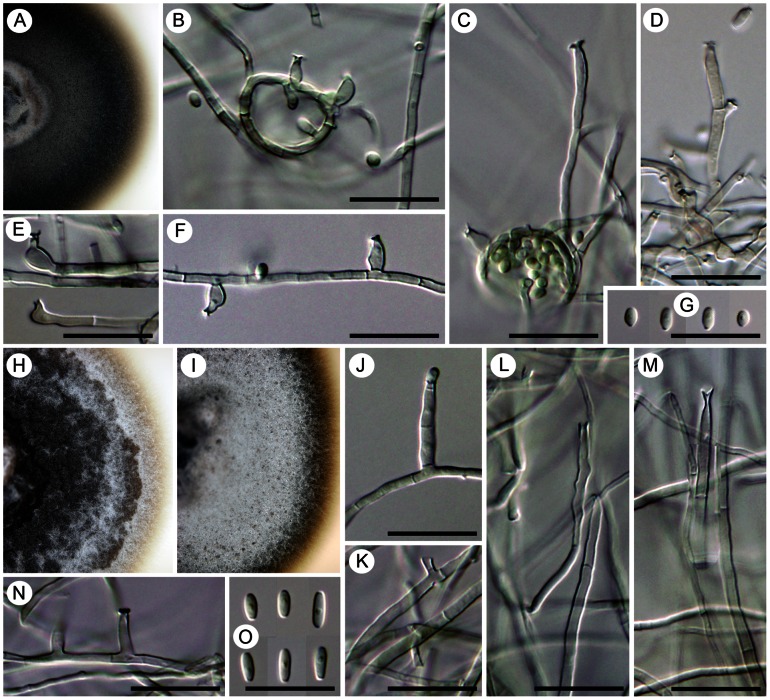
Two species of *Cyphellophora* with nonseptate conidia *in vitro*. A–G) *Cyphellophora europaea*: living culture (A), terminal or lateral phialides (B–F), and conidia (G). DIC, bar  = 20 µm. CBS 101466 (ex-type). H–O) *Cyphellophora olivacea*: living culture with a detail of aerial mycelium (H, I), terminal and lateral phialides (J, L–N), intercalary phialides (K), and conidia (O). DIC, bar  = 20 µm. CBS 122.74 (ex-type), CBS 123.74 (only O).

(de Hoog, Mayser & Haase) Réblová & Unter., comb. nov. (Fig. 8A–G).

[urn:lsid:indexfungorum.org:names: 803683]

Basionym. *Phialophora europaea* de Hoog, Mayser & Haase, Mycoses 43: 414. 2000.

Habitat. This species has been isolated most frequently from nails and skin scrapings [5], [24].

Living strains examined. CBS 101466 (ex-type), CBS 129.96.

##### Cyphellophora olivacea

(W. Gams) Réblová & Unter., comb. nov. (Fig. 8H–O)

[urn:lsid:indexfungorum.org:names: 803684]

Basionym. *Phialophora olivacea* W. Gams, Stud. Mycol. 13: 65. 1976.

Habitat. Known from various substrates including wallpaper, stalactites, dead plants, and the fruit body of a basidiomycete [19].

Living strains examined. CBS 122.74 (ex-type), CBS 123.74.

##### Cyphellophora oxyspora

**Figure 9:**
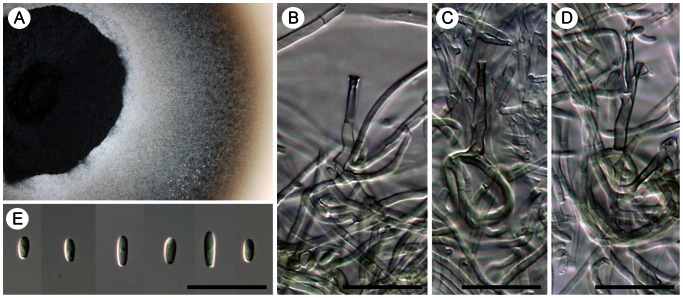
Species *Cyphellophora oxyspora* with nonseptate conidia *in vitro*. Living culture (A), phialides (B–D), and conidia (E). DIC, bar  = 20 µm. CBS 698.73 (ex-type).

(W. Gams) Réblová & Unter., comb. nov. (Fig. 9A–E)

[urn:lsid:indexfungorum.org:names: 803685]

Basionym. *Phialophora olivacea* W. Gams, Stud. Mycol. 13: 64. 1976.

Habitat. The ex-type strain was isolated from decaying leaves of *Clerodendron monahassa* (Verbenaceae) [19]. A second strain was isolated from a human toenail [11].

Living strain examined. CBS 698.73 (ex-type).

##### Cyphellophora reptans

**Figure 10:**
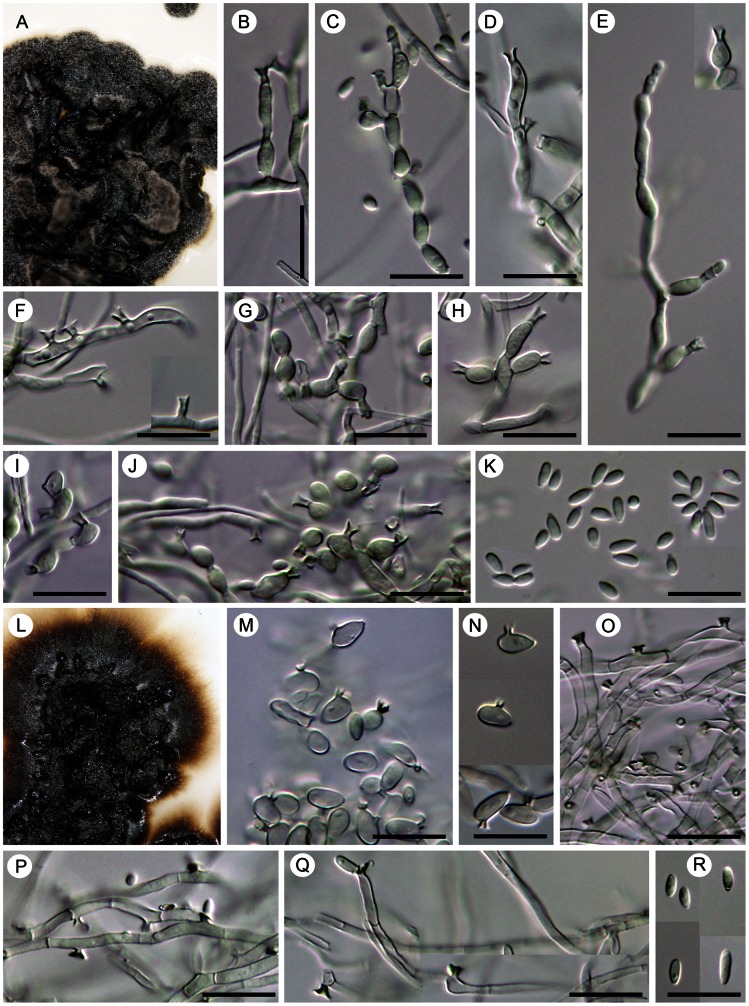
Two species of *Cyphellophora* characterized by the presence of budding and germinating cells. A–K) *Cyphellophora reptans*: living culture with a yeast-like appearance (A), lateral and terminal phialides (B, D, H), intercalary phialides (F), germinating cells (C, G, I), budding cells (J), and nonseptate conidia (K). DIC, bar  = 20 µm. CBS 113.85 (ex-type, only A), CBS 458.92. L–R) *Cyphellophora sessilis*: Living culture with sparse aerial mycelium and a yeast-like appearance (L), budding cells (M, N), terminal and lateral phialides (O, Q), intercalary phialides (P), and conidia (R). DIC, bar  = 20 µm. CBS 243.85 (ex-type).

(de Hoog) Réblová & Unter., comb. nov. (Fig. 10A–K)

[urn:lsid:indexfungorum.org:names: 803686]

Basionym. *Phialophora reptans* de Hoog, Stud. Mycol. 43: 117. 1999.

Habitat. The ex-type strain was isolated from food. Additional strains have been isolated from human nails and skin scrapings, bark, soil, and water [24].

Living strains examined. CBS 113.85 (ex-type), CBS 458.92.

##### Cyphellophora sessilis

(de Hoog) Réblová & Unter., comb. nov. (Fig. 10L–R)

[urn:lsid:indexfungorum.org:names: 803687]

Basionym. *Phialophora sessilis* de Hoog, Stud. Mycol. 43: 120. 1999.

Habitat. This species has been isolated from a variety of nutrient-poor, recalcitrant substrates including the resin of *Picea abies* (ex-type strain), styrene, and marble powder, as well as from decaying plants [24], [25]. *Phialophora sessilis* has been reported as the agent of flyspeck and sooty blotch disease [55], [56]. This species has never been documented as a cause of infections of warm- or cold-blooded animals.

Living strains examined. CBS 243.85 (ex-type), CBS 238.93.

**Figure 11:**
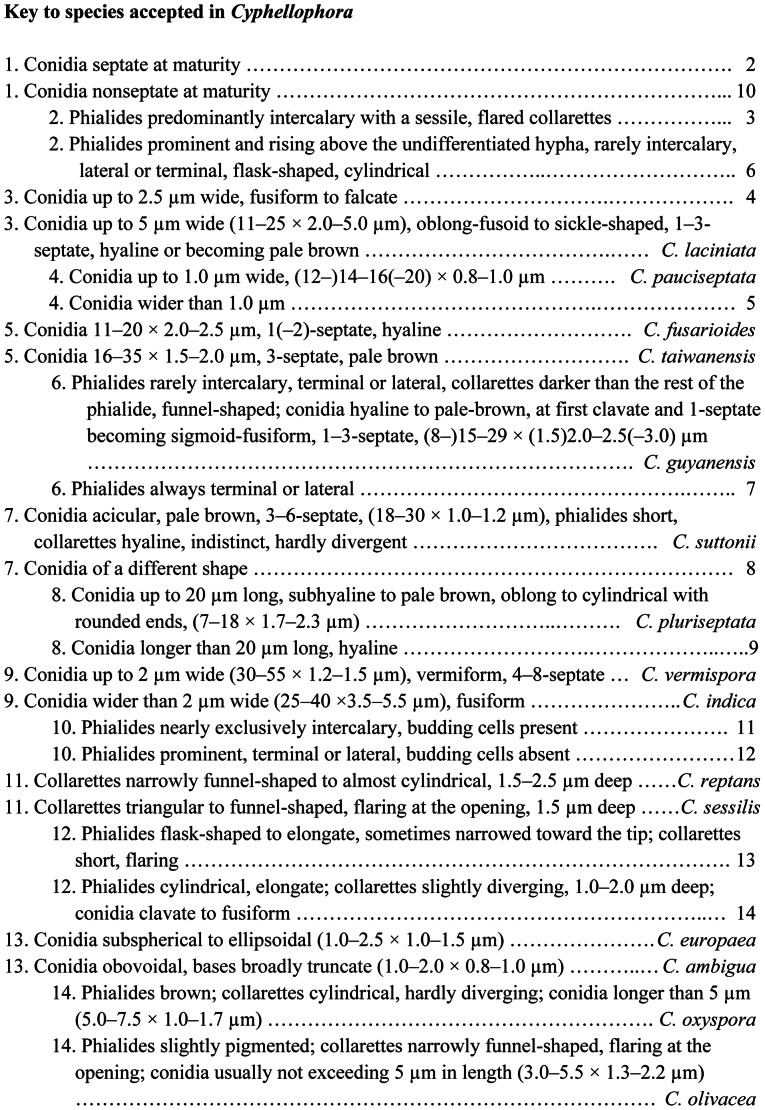
Key to species accepted in *Cyphellophora*.

Comments. Although *Cyphellophora* was originally distinguished from *Phialophora* by its septate conidia and intercalary conidiogenous cells that did not match the concept of a phialide (i.e., a discrete cell borne at the end of a short branch or laterally) [6], the concept of the genus was later broadened to include species with discrete, terminal and lateral conidiogenous cells [9]. Key to species accepted in *Cyphellophora* is shown in Fig. 11.

*Cyphellophora* comprises the type of *Kumbhamaya* M. Jacob & Bhat. Type now recognized as *Cyphellophora indica* (M. Jacob & Bhat) C. Decock, a species that resembles *C. pluriseptata* G.A. de Vries, Elders & Luykx in producing pale brown, flask-shaped phialides with elongated hyphal bases and septate, fusiform conidia. *Cyphellophora* can also be compared to *Pseudomicrodochium* B. Sutton. Two species originally classified in this genus, *C. fusarioides* (B. Sutton & C.K. Campb.) Decock and *C. suttonii*, were transferred to *Cyphellophora* by Decock *et al*. [13] based on the comparison of cultural characters and the analysis of nuc18S rDNA sequences. *Pseudomicrodochium aciculare* B. Sutton, the type species of this genus, has been collected frequently on the rotting cupules of *Castanea sativa* [57] and differs from species now treated as members of the genus *Cyphellophora* by the formation of white to pale salmon colonies, hyaline conidiogenous cells aggregating in sporodochia, and hyaline conidia *in vitro* [13]. The strain MUCL 39134 of *P. aciculare* was collected on decaying leaves and resolved as belonging to the Hypocreales [13].

The shape of the conidia of *Cyphellophora* ranges from short to elongate. Nonseptate conidia are obovoidal to ellipsoidal to clavate or slightly fusiform and are often truncate at the basal end, while septate conidia range from oblong-ovoid or oblong-fusoid to cylindrical to acicular to vermiform, and can be straight, curved or sigmoid. The *C. laciniata* clade, which is the largest group inferred in all three phylogenies (Figs. 1–3), encompasses four species with septate conidia, *viz. C. fusarioides*, *C. laciniata*, *C. suttonii*, and *C. vermispora*. Three other species of *Cyphellophora* with septate, fusiform or vermiform conidia, *viz. C. pauciseptata*, *C. guyanensis* Decock & G. Delgado, and *C. pluriseptata*, are dispersed among species with nonseptate, oblong, ellipsoidal to clavate conidia. Taxa with intercalary phialides are also not restricted to specific subclades (Fig. 1). Two species with septate conidia formed from intercalary phialides are placed in the *C. laciniata* clade (*C. laciniata*, *C. fusarioides*). A third species sharing these features, *C. pauciseptata*, is sister to the *C. laciniata* clade, while *C. guyanensis*, which produces septate conidia but seldom forms intercalary phialides, is positioned outside the core group. *Cyphellophora reptans* and *C. sessilis* are the only members of the genus with nonseptate conidia and intercalary phialides. These taxa exhibit yet another morphological peculiarity; in axenic culture they produce germinating (*C. reptans* in Fig. 10C, G) and budding cells (*C. reptans* in Fig. 10I, J and *C* *. sessilis* in Fig. 10M, N) and form yeast-like colonies (Fig. 10A, L) that are reminiscent of some *Exophiala* J.W. Carmich. (Herpotrichiellaceae) [33], [58]. Other Cyphellophoraceae produce slow growing, dark colonies *in vitro* with abundant aerial mycelium and lack budding cells. The term budding cells was first used by de Hoog *et al.* [58], in fact these cells are swollen conidia with a phialidic opening.

Information on the ecology and potential pathogenicity of the Cyphellophoraceae is incomplete. Teleomorphs are unknown for this family and all *Cyphellophora* species, except *C. europaea*, *C. laciniata*, *C. reptans* and *C. sessilis*, are known from three or fewer isolates. *Cyphellophora laciniata* [6], *C. europaea* [5], *C. oxyspora* [11], *C. pauciseptata* [11], *C. pluriseptata* [9], *C. reptans* [24], *C. suttonii* [59], and *C. vermispora* [11] have been isolated from human samples. *Cyphellophora suttonii* is also reported from animal skin (subcutaneous ear lesion) [8] and *C. fusarioides* has been isolated from pulmonary fluids [10]. Among these species, only *C. fusarioides*, *C. pauciseptata*, and *C. pluriseptata* are known solely from clinical isolates. The other species are also known from nonclinical sources. For example, strains of *C. europaea* were reported from environmental samples from humid environments (i.e., bathrooms, washing machines) [60], [61], *C. reptans* was originally isolated from food [24], a strain of *C. laciniata* was isolated from a bathroom surface, and the ex-type strain of *C. oxyspora* was isolated from decaying leaf [19]. *Cyphellophora suttonii* was also detected in soil samples, although this strain has never been sequenced [12]. Other *Cyphellophora* that have not been involved in human infections are frequently isolated from plants or nutrient-poor substrates, and two species (*C. olivacea* and *C. oxyspora*) were originally classified in the *Phialophora* sect. *Catenulatae* [19].

Although several *Cyphellophora* have been isolated from clinical sources, these fungi are not necessarily agents of infection. Nondermatophytic filamentous fungi (NDF) (i.e., species other than Arthrodermataceae) rarely infect the nails and skin of humans and animals, but it is difficult to compare the accuracy of various procedures used to determine the agents of these infections. The current standard procedure for confirming NDF as specialized for pathogenesis of nails (onychomycosis) recommends that the infecting fungus be isolated from at least two successive but temporally separate nail specimens, at least one of which is consistent for compatible filaments observed in direct KOH microscopy [62]. This is because nails with medical problems have fissures and rough surfaces that trap or contain fungi, dust and dirt which could result in the misinterpretation of the etiology of a species from a single isolation [62] (R. Summerbell pers. com.). We therefore advocate the careful evaluation of the etiology of both dermatomycoses and onychomycoses attributed to the Cyphellophoraceae before characterizing these fungi as capable of infecting nails and skin.

##### Aphanophora

Réblová & Unter., gen. nov.

[urn:lsid:indexfungorum.org:names: 803677]

Mycelium immersed and superficial, composed of branched, septate, pigmented hyphae. *Conidiophores* absent, reduced to a single conidiogenous cell. *Setae* present. *Conidiogenous cells* phialidic, intercalary, seldom terminal, multiple conidiogenous loci within the collarette; collarettes inconspicuous, hyaline. *Conidia* hyaline to subhyaline, composed of subcylindrical to cylindrical segments, marked by constrictions in the wall at the septa. Each segment divided by a secondary median septum. Conidia anastomose and undergo microcyclic conidiation in culture.

Etymology. *Aphanos* (Gk)  =  inconspicuous, referring to the inconspicuous multiple conidiogenous loci within the collarette; *phora* (Gk)  =  bearing from *pherein*  =  to bear.

Type species. *Aphanophora eugeniae* (Crous, de Hoog & H.D. Shin) Réblová & Unter.

##### Aphanophora eugeniae

**Figure 12:**
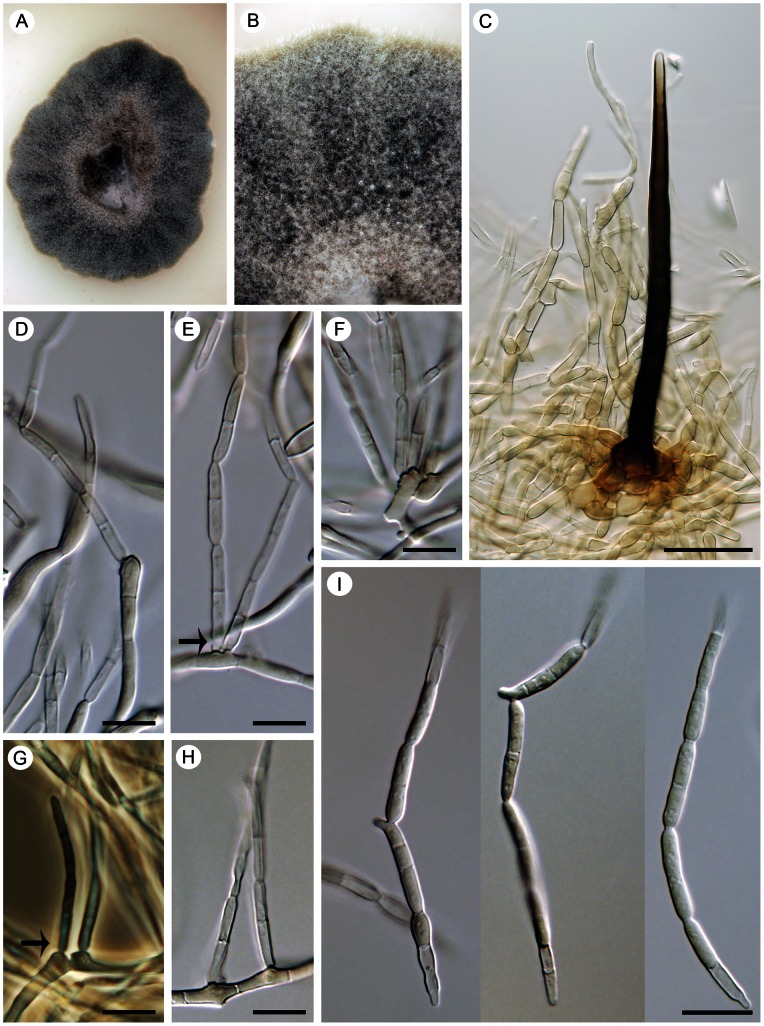
*Aphanophora eugeniae*, a foliicolous species with conidia forming long one-septate segments. Living culture with a detail of aerial mycelium (A, B), seta that occurs rarely in culture (C), phialides with multiple loci within inconspicuous collarettes (arrow indicates the collarette, D–H), and conidia undergoing microcyclic conidiation (I). DIC (C–F, H, I), PC (G), bar  = 20 µm (C), 10 µm (A, B, D–I). CBS 124105 (ex-type).

(Crous & Alfenas) Réblová & Unter., comb. nov. (Fig. 12A–I)

[urn:lsid:indexfungorum.org:names: 803678]

Basionym. *Cyphellophora eugeniae* Crous & Alfenas, Persoonia 22: 147. 2009.

Habitat. Isolated from living leaves of *Eugenia uniflora* ( =  *Stenocalyx uniflorus*) (Myrtaceae) [17].

Living strain examined. CBS 124105 (ex-type).

##### Camptophora

Réblová & Unter., gen. nov.

[urn:lsid:indexfungorum.org:names: 803679]

Mycelium immersed and superficial, composed of branched, septate, pigmented hyphae. *Conidiophores* absent, reduced to a single conidiogenous cell. *Conidiogenous cells* phialidic, intercalary, seldom terminal, proliferating percurrently, single conidiogenous locus within the collarette; collarettes distinct, hyaline, narrowly funnel-shaped, hardly divergent. *Conidia* hyaline, subhyaline to light brown, fusiform or sickle-shaped, septate, constricted at the septa. Conidia anastomose and undergo microcyclic conidiation in culture.

Etymology. *Kamptós* (Gk)  =  curved, referring to the curved conidia; *phora* (Gk)  =  bearing from *pherein*  =  to bear.

Type species. *Camptophora hylomeconis* (Crous, de Hoog & H.D. Shin) Réblová & Unter.

##### Camptophora hylomeconis

**Figure 13:**
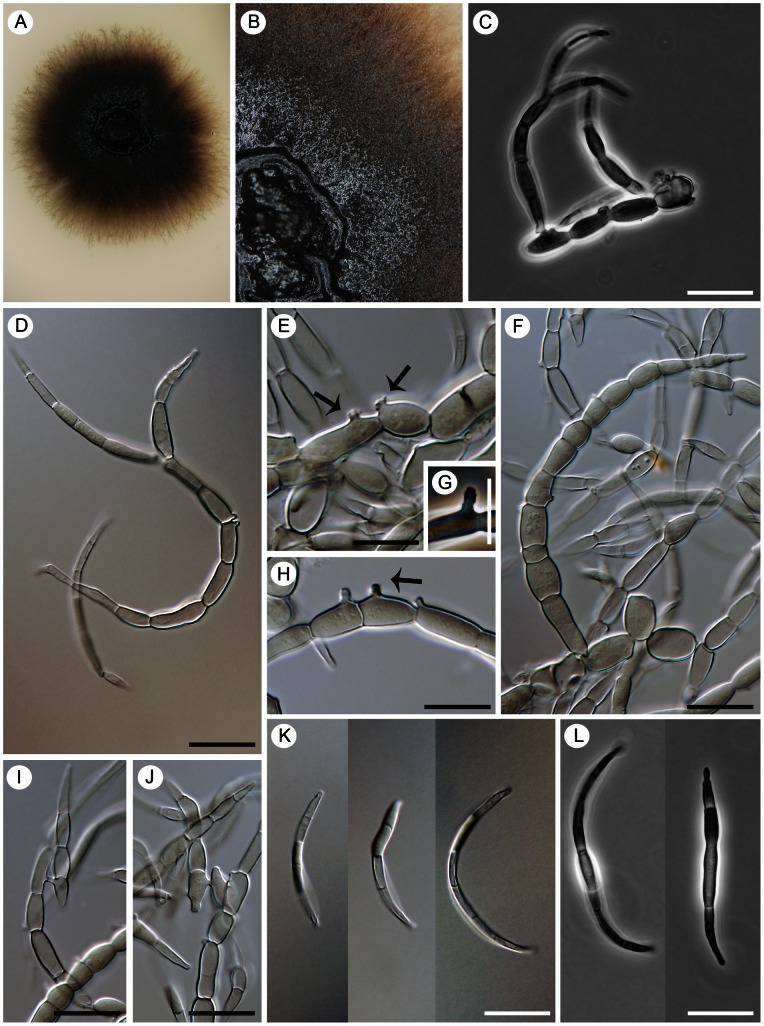
*Camptophora hylomeconis*, a foliicolous species with sickle-shaped septate conidia. Living culture with a detail of the center of the colony having a yeast-like appearance (A, B), intercalary phialides with conidia (C, D), a detail of conspicuous phialidic loci that can proliferate percurrently above the collarette (arrows indicate the proliferation, E–F), anastomosing conidia (I, J), and sickle-shaped or sigmoid septate conidia (K, L). DIC (D–F, H–K), PC (C, G, L), bar  =  10 µm. CBS 113311 (ex-type).

(Crous, de Hoog & H.D. Shin) Réblová & Unter., comb. nov. (Fig. 13A–L)

[urn:lsid:indexfungorum.org:names: 803680]

Basionym. *Cyphellophora hylomeconis* Crous, de Hoog & H.D. Shin, Stud. Mycol. 58: 200. 2007.

Habitat. Isolated from living leaves of *Hylomecon vernalis* (Ranunculaceae) [14].

Living strain examined. CBS 113311 (ex-type).

**Figure 14:**
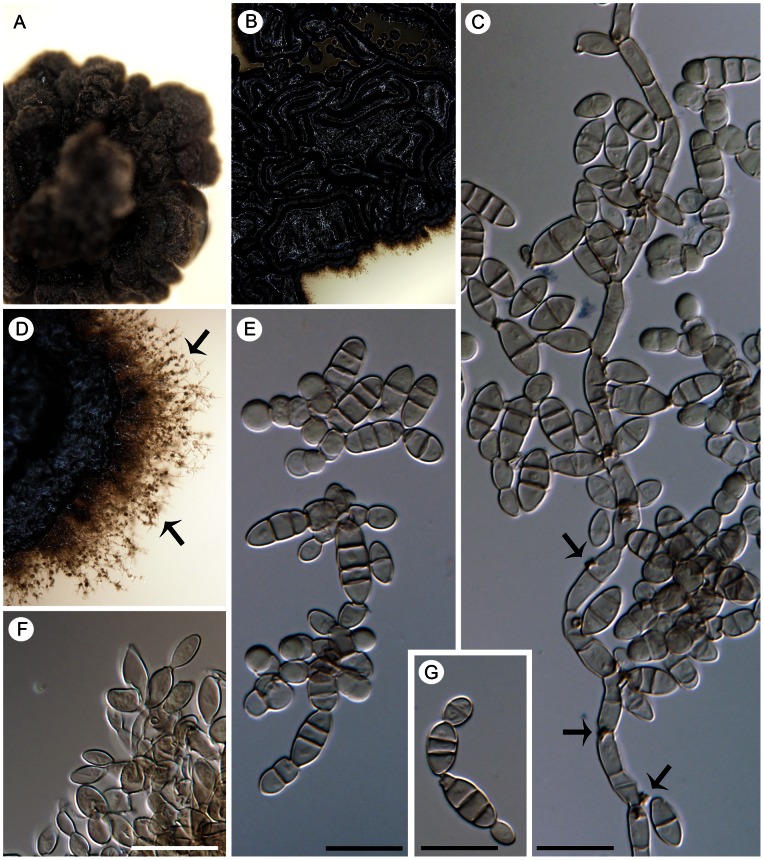
*Exophiala eucalyptorum*, a dematiaceous hyphomycete with rare hyphal growth *in vitro*. Living culture with a detail of the colony with sparse aerial hyphae (A) and moist, cerebriform surface (B), hyphae from the margin of the colony with a phialidic opening in every cell (C, D), nonseptate conidia (F; 21 days), and germinating several-septate conidia (arrows indicate proliferating phialidic openings, E, G; 3 months). DIC, bar  = 10 µm. CBS 121638 (ex-type), WUC 637 (only B, D).

**Figure 15:**
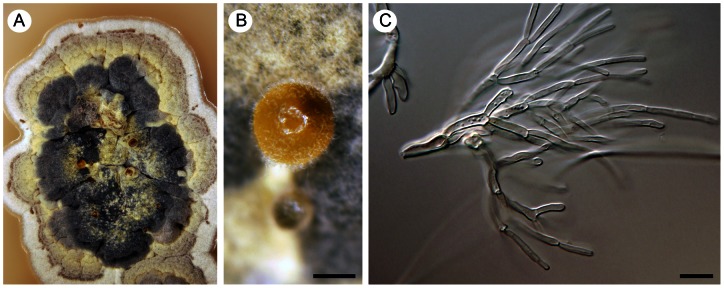
*Vonarxia vagans*, a foliicolous hyphomycete with blastic conidiogenesis. Living culture with several conidiomata visible in the center of the colony (A) and a detail of the conidioma with conidia extending from the surface of the yellow gelatinous matrix (B), branched mycelium forming conidia at the tips (C). DIC, bar  =  1 mm (B), 10 µm (C). CBS 123533 (ex-type).

Comments. Although not closely related, *Aphanophora eugeniae* and *Camptophora hylomeconis* group within the Chaetothyriaceae with *Exophiala eucalyptorum* (Fig. 14) and *Vonarxia vagans* (Fig. 15) in all three phylogenies (Figs. 1–3). Based on molecular data and their distinctive morphologies, these taxa are excluded from *Cyphellophora* and introduced as the type species of separate genera.

*Aphanophora, Camptophora* and *E. eucalyptorum* grouped constantly together in the ITS, ITS-β-tubulin and four-gene phylogenies and form predominantly intercalary phialides with minute or inconspicuous hyaline collarettes. Their conidia undergo frequent microcyclic conidiation (Figs. 12I; 13C, D) in axenic culture (and as budding and germinating cells in *E. eucalyptorum*, Fig. 14E, G). The main differences between *Aphanophora* and *Camptophora* lie in the morphology of phialidic loci and presence or absence of conidial segments. In *Aphanophora* one to three phialidic openings are formed in each intercalary phialide; the collarettes are inconspicuous, and the multiple conidiogenous loci become swollen and may appear subdenticulate [17]. The conidia, which are composed of several constricted segments with a median secondary septum, closely resemble those of *V. vagans* [63] and anamorphs of *Ceramothyrium* [64], [65]. In contrast, *C. hylomeconis* produces conspicuous phialidic openings with single conidiogenous loci (rarely two loci) within the collarette that proliferate percurrently. The conidia are (1–)3–5-septate, fusiform to sickle-shaped with constricted cell walls, but the conidial segments observed in *Aphanophora* are not formed.

##### Knufia peltigerae

(Fuckel) Réblová & Unter., comb. nov.

[urn:lsid:indexfungorum.org:names: 803681]

Basionym. Trichosphaeria peltigerae Fuckel, Jb. nassau. Ver. Naturk. 27–28: 25. 1874 (1873–74).

≡ *Capronia peltigerae* (Fuckel) D. Hawksw., Syst. Ascom. 6: 120. 1987.

Habitat. The ascomata of *K. peltigerae* occur on thalli of species of *Peltigera* [66], [67], [68].

Comments. In the ITS and four-gene phylogeny, *Capronia peltigerae* grouped among species of the genus *Knufia*. Our results are in agreement with Untereiner *et al*. [68], who positioned this species outside the Herpotrichiellaceae among rock-inhabiting strains and other species currently referred to *Knufia* [37]. In axenic culture, *K. peltigerae* exhibits only filamentous growth and remains sterile. No conidial anamorph has been observed around the ascomata of this species on natural substrates [67], [68]. *Knufia peltigerae* represents the only member of the genus with a known teleomorph. In the morphology of its dark, setose ascomata that contain saccate bitunicate asci with hyaline, septate ascospores, it is similar to the known epiphyllous teleomorphs of the Chaetothyriaceae [38], [64] and Trichomeriaceae [39].

##### Phaeococcomyces catenatus

(de Hoog & Herm.-Nijh.) de Hoog, Taxon 28: 348. 1979.

≡ *Phaeococcus catenatus* de Hoog & Herm.-Nijh., Stud. Mycol. 15: 126. 1977.

 =  *Exophiala placitae* Crous & Summerell, Fungal Planet 17: 1. 2007.

Habitat. The ex-type strain of *P. catenatus* was isolated from the air [58] and the ex-type strain of *E. placitae* formed pycnothyria on leaves of *Eucalyptus placita* [69].

Comments. In the ITS and four-gene phylogenies (Figs. 1, 3) the ex-type strain of *P. catenatus* (CBS 650.76) grouped consistently in a strongly supported, monophyletic clade with the ex-type strain of *E. placitae* (CPC 13707). Based on the morphological similarities of these species *in vitro*, *E. placitae* is reduced to synonymy of *P. catenatus*. Both fungi produce abundant globose, budding cells in axenic culture that are pale brown, 0-2-septate, and give rise to new identical cells from 1–6 loci. The cells adhere in torulose, branched or unbranched chains. Hyphae are absent although some short, torulose fragments may recall hyphal growth. *Phaecoccomyces catenatus* was previously known only *in vitro*. The strain CPC 13707 (herbarium sample CBS H-19922) represents a first documented case of occurrence of this fungus on natural substratum [69]. *In vivo* this species forms dark, flat, centrally ostiolate pycnothyria. Conidia are pale brown, globose, 1-celled, and forming up to six torulose arms composed of 5–8 cells, with each cell forming short lateral branches composed of several cells.

## Discussion

### Chaetothyriales

The Chaetothyriales encompass fungi with small, typically setose ascomata, fissitunicate, thick-walled, sessile asci, and hyphomycetous anamorphs and synanamorphs. Ecologically, the order comprises taxa occurring on a variety of substrates, and it includes both saprobic species and opportunistic pathogens of humans and animals. Phylogenetic analyses of five genes supported the recognition of the Chaetothyriales together with the Pyrenulales and Verrucariales as members of the Chaetothyriomycetidae, one of the three subclasses of the Eurotiomycetes [16], [70].

Only two of the eight families distinguished in the order by Barr [71], [72], the Chaetothyriaceae and Herpotrichiellaceae, have been shown to be closely related to the Eurotiales [73]. The third family Coccodiniaceae, although sometimes still accommodated in the order [74], is more closely allied to the Dothideomycetes [73]. The monotypic Trichomeriaceae, accepted as a part of the Chaetothyriaceae in our study, were introduced recently [39]. Based on molecular and DNA structural data, we introduce the new family Cyphellophoraceae.

### Cyphellophoraceae

The Cyphellophoraceae is a phylogenetically distinct lineage that contains *Cyphellophora laciniata*, the type species of the genus, six other *Cyphellophora*, and six species treated previously in *Phialophora*. Our conclusions are in agreement with those of de Hoog *et al*. [5], Untereiner *et al*. [18], and Feng *et al*. [11] who recognized a strongly supported clade separate from the Herpotrichiellaceae containing numerous strains of *C. europaea*, *C. reptans* and *C. sessilis*.

The core of the family contains four species (*C. laciniata*, *C. fusarioides*, *C. suttonii*, *C. vermispora*) that are consistently resolved as sister to the morphologically similar taxon, *C. pauciseptata* in the ITS and ITS-β-tubulin phylogenies (Figs. 1, 2). *Cyphellophora pauciseptata* and *C. fusarioides* are the only species isolated from clinical sources and they are resolved as the members of the same clade in the four-gene analysis (Fig. 3).

Species of *Cyphellophora* are distinguished from *Phialophora verrucosa* (Herpotrichiellaceae) by their cylindrical or flask-shaped elongate or intercalary phialides with hyaline or slightly pigmented collarettes that are usually narrowly funnel-shaped with flaring opening or almost cylindrical and hardly divergent. In contrast, *P. verrucosa* possesses flask-shaped phialides with broadly funnel-shaped or vase-shaped collarettes with flaring openings that are always deeply pigmented and conspicuously darker than the lower part of the phialide. Ten species of *Cyphellophora* were isolated from skin, nail or other clinical specimens from humans and animals. However, they are clinically distinct from opportunistic pathogens of humans and warm- or cold-blooded animals [4], [5], [14], [24] centered on *P. verrucosa* and other Herpotrichiellaceae that cause chromatoblastomycoses and phaeohyphomycoses. Members of the Herpotrichiellaceae are also known as saprobes on decaying plant material [75], [76]; a number of these fungi have been isolated from nutrient-poor substrates in extreme environments and are known as rock-inhabiting fungi [77].

Teleomorphs are unknown in the Cyphellophoraceae. The Herpotrichiellaceae accommodates the teleomorph genus *Capronia* Sacc., which is linked with *Phialophora* and a number of hyphomycetous anamorphs and synanamorphs that exhibit yeast-like growth in axenic culture. The majority of these anamorphic fungi are species of *Cladophialophora* Borelli, *Exophiala* J.W. Carmich., *Fonsecaea* Negroni, and *Rhinocladiella* Nannf. [33], [78], [79], [80], [81], [82], [83].

### Chaetothyriaceae

The Chaetothyriaceae were resolved as a robust heterogeneous clade consisting of two subclades in the ITS (99/1.0) and four-gene phylogenies (90/1.0) (Figs. 1, 3). This family, which is also supported by the presence of evolutionarily conserved motifs (M2 and M3) in the ITS2, encompasses saprobic, rock-inhabiting, lichenicolous, epiphytic or biotrophic epiphyllous fungi [37], [38], [39], [64], [65], [68], [84], [85], [86], that are rarely isolated from clinical samples [87].

The monotypic family Trichomeriaceae [39] is treated here as a part of the Chaetothyriaceae. This lineage is positioned within a large subclade (100/1.0 in ITS and four-gene analyses) that also includes species of *Knufia, Brycekendrickomyces acaciae, Cladophialophora proteae*, and *Phaeococcomyces catenatus*. The similarities of the dark, nonstromatic ascomata, sessile, saccate, fissitunicate asci and hyaline, septate ascospores of *Knufia peltigerae* and species of *Trichomerium* [39] are remarkable. Species of *Knufia*, except *K. peltigerae*, have not been linked to teleomorphs. The genus comprises dematiaceous fungi forming sporodochia on the host that contain conidia and multicelullar bodies. In axenic culture, these species produce thallic and blastic conidia from undifferentiated hyphae as well as from phialides; they rarely form endoconidia [37], [88]. According to Tsuneda *et al.* [37] and Gueidan *et al*. [77] the nuc28S sequence and culture (CBS 268.34) of *Glyphium elatum*, which grouped in *Knufia*, are incorrectly determined and we follow their preliminary placement and identification of this strain as *Coniosporium* sp.

*Phaeococcomyces catenatus* produces exophiala-like colonies in axenic culture, while cheirospores are formed in pycnothyria on the host (CPC 13707 as *Exophiala placitae*) [69]. *Metulocladosporiella* [36] is characterized by its dematiaceous, macronematous conidiophores that form terminal, branched, polyblastic, sympodial conidiogenous cells and conidia that adhere in chains. The monotypic genus *Brycekendrickomyces* Crous & M.J. Wingf. grouped on a basal branch in this robust subclade. It was isolated from living leaves of *Acacia auriculiformis* and produces macronematous, dematiaceous conidiophores with one to several polyblastic conidiogenous cells formed at the tips and hyaline, ellipsoid conidia aggregated in slimy heads [17].

The second subclade included in the Chaetothyriaceae is well-supported in ITS, ITS-β-tubulin and four-gene analyses (Figs. 1–3) and comprises three species of *Ceramothyrium*, the *Vonarxia*-group (including *Aphanophora, Camptophora*, and *Exophiala eucalyptorum*) and *Chaetothyrium brischofiicola*. Members of this clade are filamentous dematiaceous fungi, although the filamentous nature of *E. eucalyptorum* is inconspicuous. In axenic culture, this species forms immersed, septate hyphae at the margins of the colony (Fig. 14D) with intercalary phialides arising from nearly each cell (Fig. 14C). Teleomorphs of *Ceramothyrium*, *Chaetothyrium* and *Phaeosaccardinula* differ from *Knufia* and *Trichomerium* in possessing a thin pellicle that covers the ascomata and merges with subiculum on the surface of the host.

With the exception of *Phaeosaccardinula fici* [38], the members of the *Vonarxia*-group (Figs. 1–3) are anamorphic fungi. These species are isolated exclusively from living plants or plant litter and are not involved in human or animal infections. Morphologically, this clade is highly variable and includes species producing blastic conidia as well as conidia from phialidic openings. *Aphanophora*, *Camptophora* and *E. eucalyptorum* produce intercalary phialides. Inconspicuous phialidic openings can also be formed on mature conidia, which anastomose and undergo frequent microcyclic conidiation. The phialidic loci can proliferate percurrently above the collarette in the latter two taxa (Figs. 13E, G, H; 14C). Conidiogenesis in *V. vagans* is blastic (Fig. 15C); hyaline conidia consisting of three upper arms on a short main axis are formed sympodially from one to three different loci at the top of a doliform conidiogenous cell [17], [63]. In fact, each arm is formed by several 1-septate segments marked by constrictions in the wall at the septa. A secondary septum is positioned in the middle of each segment. Anamorphs experimentally linked with *Ceramothyrium* were originally described in *Stanhughesia* Constant. [65] and resemble *V. vagans* in conidiogenesis and conidium morphology. The hyaline blastic conidia of *Ceramothyrium* are L-shaped, consisting of a main septate or nonseptate axis with one or two laterally oriented septate arms composed of nonseptate or one-septate segments. Cylindrical-elongate conidia divided into septate segments also occur in *Aphanophora*.

Two taxa originally classified in the Chaetothyriaceae, *Ceramothyrium thailandicum* and *Phaeosaccardinula fici* [38] were placed in different clades in the four-gene and ITS analyses. In the multilocus analysis, *Ceramothyrium thailandicum* grouped with two other *Ceramothyrium* in a weakly supported clade (Fig. 3), but in the preliminary ITS analysis it was positioned outside the Chaetothyriaceae (not shown). The main differences between the ITS2 of *C. thailandicum* (strain MFLUCC 10-0079) and *C. carniolicum* (strain CBS 175.95), is that the sequence of the former species includes a H3A helix formed from eleven bp and five other nucleotides in the end loop. This type of helix was observed only in the Cyphellophoraceae, Herpotrichiellaceae, and selected Chaetothyriaceae (i.e., *V. vagans*, *Metulocladosporiella* and *Phaeococcomyces*). Because the placement of *C. thailandicum* in our preliminary ITS analysis does not agree with the study of Chomnunti *et al*. [38], this sequence was not included in the final ITS analysis.

Available sequences of *Phaeosaccardinula fici* (Chaetothyriaceae) (ITS HQ895840; nuc28S HQ895837) [38] derived from the same strain MFLUCC 10-0080 were positioned in different clades. In the preliminary ITS analysis (not shown) this species was positioned as sister to the Chaetothyriaceae. In the four-gene phylogeny (Fig. 3), it was resolved as sister to *Vonarxia vagans*. Nag Raj [63] observed several immature ascomata on a stroma of *V. vagans* on the host in the type material, LPS 12280 as *Kazualia vagans* (Speg.) Nag Raj, quoting Spegazzini's handwriting on the label. The suggested link between the two morphs still needs to be confirmed experimentally. However, the grouping of *P. fici* with anamorphic *V. vagans* in one clade in the four-gene phylogeny suggests such connection and the existence of a teleomorph for the later taxon. Considering the contradictory phylogenetic relationships and the probable anamorph-teleomorph link between *Vonarxia* and *Phaeosaccardinula*, only the nuc28S sequence was included in our study.

### Secondary structure of ITS

Comparison of 2D structures of ITS1 and ITS2 of members of the Chaetothyriales confirmed three major phylogenetic groups at the family level. Within this order we recorded 45 and 64 conserved nucleotides in the three helices of the ITS1 (Fig. 5) and four helices of ITS2 (Figs. 6, 7), respectively. These homologous characters are unevenly distributed in hairpin loops and served as a backbone of the multiple sequence alignment.

The three evolutionary motifs (Table 1) observed in the primary ITS2 sequence were mapped on the ITS phylogram (Fig. 1) and 2D structures of ITS2 (Figs. 6, 7). The M1 motif with three recorded deviations is repeated in all members of the Chaetothyriales. The other two motifs identified in the H3 helix of ITS2 characterize certain clades. The M2 motif is unique for the Cyphellophoraceae (UCUG) and the Herpotrichiellaceae (CCUG), but varies slightly within the Chaetothyriaceae (UCCG), i.e., CCUA in *Ceramothyrium carniolicum*, UCAG in *Cladophialophora proteae* and UCCC(U) among species of *Trichomerium*. The M3 motif is considered one of the hallmarks of the ITS2 secondary structure [29] and it may indicate family or even higher taxonomic relationships. It defines the Cyphellophoraceae (UGUA), Herpotrichiellaceae (GGUA) and some Chaetothyriaceae (GGUG). The subclade containing the *Vonarxia-*group, *Ceramothyrium* and *Chaetothyrium* is delimited by the GGUG motif and is accepted as the Chaetothyriaceae s. str. It is sister to a robust subclade within the Chaetothyriaceae that includes three different M3 motifs (Fig. 1). The most common motif, GGUG, occurs in the majority of taxa and two unique motifs, UUCG and GAUA, are found in *Trichomerium* and *Phaeococcomyces*, respectively. This second subclade may represent several families but we think it is more likely that the M3 motif will be shown to deviate among genera within a single family.

*ITS1*. – An experimental 3D structure of ITS1 does not exist but study of the 2D structure of the ITS1 in eukaryotes using comparative methods reveals an open loop with multiple helices [89], [90], [91], [92]. One of these helices (H2, Fig. 5) used to define species complex groups [93], [94] was detected in fungi by Réblová and Winka [95] who then employed the 2D structure of H2 to delimit groups of anamorphs linked with *Chaetosphaeria* of the Chaetosphaeriaceae.

The consensus 2D structure of ITS1 of members of the Chaetothyriales determined in this study comprises three helices (H1−H3) and a highly variable region at 3′-end (*ca* 60−70 nt) containing many insertions and deletions. We recorded significant variation among *Cyphellophora* in one of the two CBCs in H1 (Fig. 5) (i.e., C = G in the Herpotrichiellaceae, A-U in the *C. laciniata* clade, G = C in other Cyphellophoraceae). In five species with G = C substitution we observed another change in the primary sequence that resulted in a minor change in the stem structure. Specifically, *C. ambigua*, *C. guyanensis*, *C. olivacea*, *C. pluriseptata* and *C. reptans* possess a ‘C’ insertion on the 5′side of the helix H1 that causes a rise of the ‘U’ bulge (Fig. 5B) such that the ‘C’ insertion after U leads to a preferred formation of a canonical C = G pair (followed by the CBC A-U → G = C) instead of forming a wobble pair U/G characteristic of almost all members of the Chaetothyriales.

We observed a G/G non-canonical pair in the right arm of helix H3 in all members of the Cyphellophoraceae (Fig. 5B), it always occurs after the third base pair. This mismatch does not occur in the Herpotrichiellaceae (where 2×2 symmetrical loop exists) and other members of the Chaetothyriales. In two species formerly included in *Cyphellophora* (*Aphanophora eugeniae* and *Camptophora hylomeconis*) it changes to a wobble pair (G/U).

Considering the number of CBCs and hCBCs in the three helices of ITS1 and comparing them to the four helices of ITS2, the 2D structure of ITS1 seems more conservative among members of the Herpotrichiellaceae, Cyphellophoraceae and *Vonarxia*-group.

*ITS2*. – As with the ITS1, the 3D structure of ITS2 has not been determined. However, the 2D structure of the ITS2 has been investigated [30], [91], [96], [97], [98], [99]. The number of four helices represents one of the hallmarks and is universal among eukaryotes. Additional helices may be positioned on the ring structure anywhere between helices 1−4 in different groups of organisms [29]. This feature was observed in the Chaetothyriales. A short helix labeled H3A (Figs. 6, 7) was recognized in the Herpotrichiellaceae, Cyphellophoraceae and three Chaetothyriaceae. The basal part of H3A consists of three bp with co-evolving nucleotides (CBCs), while the upper part is highly variable and includes an internal loop and additional base pairs.

Only the Herpotrichiellaceae possess four basal bp in helix H1 (Fig. 6). In the Cyphellophoraceae and *Vonarxia*-group, two non-CBCs changes occur in the second and third bp resulting in the disruption of the duplex (Fig. 7B). The H1 helix of these taxa is shorter with longer adjacent one-stranded regions. Helices H2 and H3 of ITS2 are most variable among the Cyphellophoraceae. The pyrimidine–pyrimidine mismatch of H2 was not present in all species and the predicted models of 2D structure of ITS2 revealed this region only in *C. europaea* (U/U, U/C), *C. oxyspora* (U/U, C/C) and *C. reptans* (C/C). Although helices H1 and H4 of the ITS2 were considered the most variable regions at species levels among eukaryotes [29], these regions are more or less conserved within the Chaetothyriales.

### Changes in CBCs and hCBCs as a tool to improve alignments and to study evolutionary history of ITS1 and ITS2

Examination of all positions in ITS1 (H1–H3) and ITS2 (H1–H4) revealed that some pairs with double-sided (CBC) and one-sided (hCBC) substitutions uniquely characterize certain clades and represent non-homoplasious synapomorphies within the Chaetothyriales (Figs. 4, 6, 7). In H1–H3 of ITS1 only five base pairs that always display CBCs and thirteen that contained hCBCs or both CBCs/hCBCs were discerned. Of these, two CBCs and one hCBCs are unique for the Cyphellophoraceae. Ten base pairs in the entire ITS2 molecule displayed both CBCs and hCBCs, while only six pairs displayed CBCs; only eight of the former and three of the latter were recorded in the conserved regions of H2 and H3 helices. Of these, only one CBC and one hCBC are unique to the Cyphellophoraceae and one CBC characterizes the *Vonarxia*-group in the Chaetothyriaceae s. str. Another base pair of the five in H3, where non-CBCs were observed, is unique to the *Vonarxia*-group.

We observed that CBC and hCBC substitutions are not equally distributed in ITS of the lineages resolved in the Chaetothyriales. Caisová *et al*. [97] proposed that CBC and hCBC substitutions have evolved independently and thus hCBCs do not contribute to the origin of CBCs (i.e., hCBCs are not the intermediate steps between G = C ↔ G/U ↔ A-U). Since a G = C pair with three hydrogen bonds is more stable than A-U and G/U pairs with just two hydrogen bonds [100], G = C contributes more to the folding and stability of RNA transcripts than A-U and wobble G/U pairs, even though an important role in stability of RNA molecules is also played by stacking interactions [101]. Caisová *et al*. [97] suggested that two-sided substitutions leading to the formation of canonical G = C, C = G pairs, via short-lived non-paired intermediates (i.e., N = N ↔ N/N ↔ N-N), may occur in organisms undergoing specialization to certain habitats (e.g. higher temperatures). The rapidly evolving hCBCs substitutions, which occur more frequently in taxa, may also facilitate faster ecological adaptations of organisms followed by changes in morphology. These adaptations may involve changes of substrate.

This hypothesis is supported in our study among phylogenetically closely related members of the Chaetothyriales. For example, hCBCs and pairs with non-compensating changes in conserved areas of H2 and H3 of the ITS2 vary significantly within *Cyphellophora.* We observed five pairs in the H2 that show canonical G = C, C = G pairs in majority *Cyphellophora*, but several species are characterized by a wobble G/U, U/G pairs. Non-CBCs pairs occur in *Cyphellophora* with lower frequency than the hCBCs; in three *Cyphellophora* with non-septate conidia we discerned two non-CBCs substitutions (the pyrimidine-pyrimidine mismatch of H2) while other species possess canonical A-U, G = C pairs at these positions. In contrast, among members of the Chaetothyriales the H3 of ITS2 contains five base pairs with non-CBCs and three with hCBCs. Two of the former and two of the latter are the sources of the greatest infrageneric variability of *Cyphellophora*. In addition, we distinguished two pairs exhibiting CBCs, hCBCs and non-CBCs (both in H3) in ITS1 that occur only in the Cyphellophoraceae and characterize individual species. We assume the occurrence of non-CBCs and hCBCs substitutions in *Cyphellophora* at certain base pairs indicate broad ecological adaptations that are reflected as saprobic (considering a wide range of substrates) or parasitic life-styles of these species.

Two newly segregated monotypic genera in the *Vonarxia*-group, *Aphanophora* and *Camptophora*, differ from *Cyphellophora* in five (H2) and eight (H3) pairs showing CBCs, hCBCs and non-CBCs substitutions in ITS2, respectively. Six pairs with changes were observed between *Cyphellophora* and both segregated genera (i.e., two CBCs, two hCBCs and two non-CBCs). Six different substitutions (i.e., two CBC, three hCBCs and one non-CBC) occur only between *Cyphellophora* and *Camptophora* and only one base pair with hCBC is unique between *Cyphellophora* and *Aphanophora*.

The CBC species concept, which has been used to delimit biological species, is based on co-evolution of nucleotides in the most conserved helices (H2 and H3) of the ITS2 [30], [31], [102], [103], [104]. Since even a single CBC in H2 and H3 of the ITS2 indicates sexual incompatibility, the original hypothesis defined a CBC clade as including organisms lacking CBCs in these conserved helices that differ from other CBC clades by as little as a single CBC. In contrast, CBCs in H1 and H4 of ITS2, and hCBCs in H2 and H3 may still allow mating between two organisms and therefore are less useful in species delimitation [31]. Also, it is important to note that the absence of CBC between two organisms does not indicate that they belong to different species [72]. CBC clades usually fall into one or more Z clades encompassing groups of organisms producing compatible gametes that can form zygotes [31], but which are separated by various pre- and postzygotic isolation mechanisms. Therefore, the Z clades rather than the CBC clades contain ‘biological species’ or represent a diverging population of one to several morphotypes capable of interbreeding.

The CBC hypothesis has been tested in Chlorophyta and representatives of the Fagales [31], [105], [106] and also by employing up to 100,000 2D models from the ITS2 database [52], [107], [108], [109]. In-depth analysis of the ITS2 of members of the Ulvales (Chlorophyta) revealed a number of discrepancies between the proposed CBC concept and the evolution of the ITS2 [97] and it was concluded that careful analysis of ITS2 evolution and phylogeny is required before species are proposed based on the presence or absence of CBCs.

Because our data sets contained a limited number of strains of each species we could not investigate the CBC species concept in each clade at a level that would resolve differences between closely related taxa or indicate cryptic species. However, recording all observed substitutions and mapping them onto the 2D structure of ITS1 and ITS2 allowed us to significantly improve the alignment of ITS sequences of members of the Chaetothyriales.

**Figure 16:**
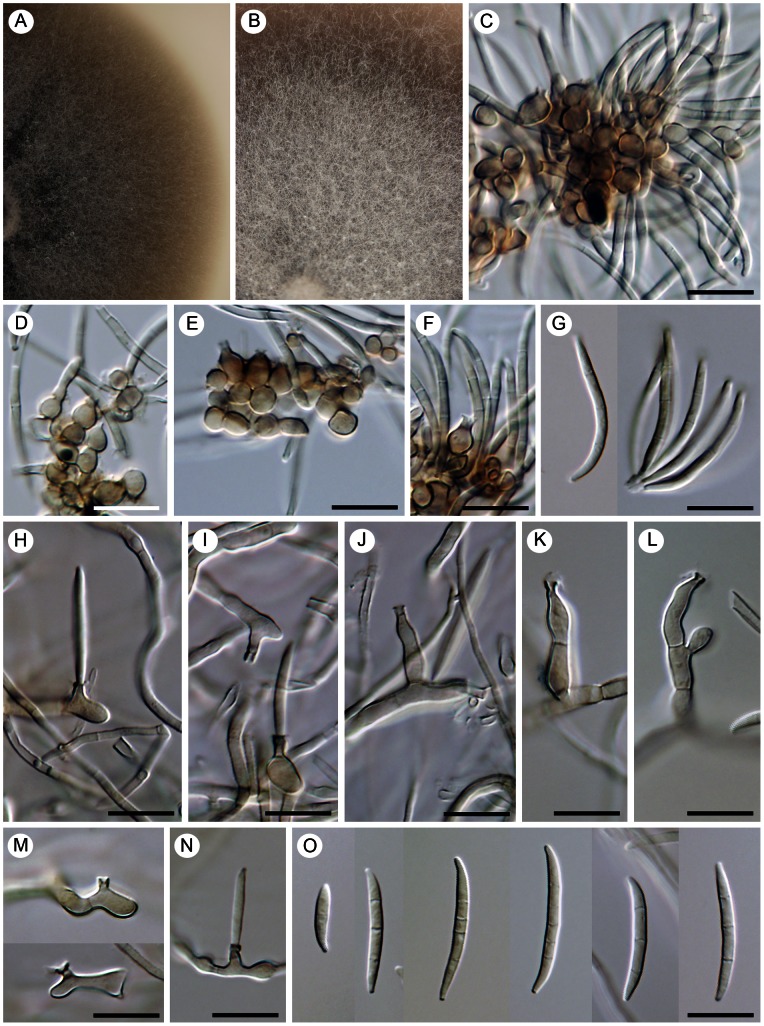
Morphological variability of *Cyphellophora guyanensis*. A, B, H–O) the ex-type strain of *Cyphellophora eucalypti*: living culture with a detail of aerial mycelium (A, B), intercalary phialides (H, I, M, N), lateral and terminal cylindrical phialides (J–L), and septate, falcate conidia (O). DIC, bar  =  10 µm. CBS 124764 (ex-type). C–G) the ex-type strain of *Cyphellophora guyanensis*: lateral flask-shaped phialides aggregated in groups on undifferentiated hyphae (C–F) and conidia (G). DIC, bar  =  10 µm. MUCL 43737 (ex-type).

The existence of two Z clades within *Cyphellophora guyanensis* (Fig. 16) is suggested by differences in the 2D structure of the conserved part of ITS2 of the isolates found in this lineage. This clade contained sequences of five strains; four of *C. guyanensis,* including the ex-type strain (MUCL 43737), and the ex-type strain of *C. eucalypti* Cheewangkoon & Crous (CBS 124764). These species are morphologically similar, and *C. eucalypti* has been treated recently as a synonym of *C. guyanensis* based on the comparison of sequence data [11]. However, Feng *et al*. [11] did not include the ex-type strain of *C. guyanensis* in their analyses. In our ITS and ITS-β-tubulin phylogenies, all five strains formed a well-supported CBC clade with two Z clades, each of which contained an ex-type strain. In the ITS phylogeny (Fig. 1), the ex-type strain of *C. eucalypti* (CBS 124764) was positioned as separate from the subclade that included four strains of *C. guyanensis*. In the ITS-β-tubulin analysis (Fig. 2) the CBC clade was again divided into two subclades; one containing the ex-type strain of *C. guyanensis* (MUCL 43737) and the other comprising the remaining isolates of this species. We observed the following differences in the ITS1 and ITS2 between the ex-type strain of *C. guyanensis* and the other members of this clade: 1) the last two bp differ in the right arm of helix H3 of ITS1 of MUCL 43737; the last bp is missing due to a non-CBC change (U-A → U/C) resulting in a longer loop and the second last bp is changed to a wobble pair (C = G → U/G), (2) the tenth bp with canonical G = C pair of MUCL 43737 is changed to a wobble pair G/U in helix H2 of ITS2, and 3) MUCL 43737 has the longest hairpin loop (i.e., 5 nt *vs*. 3 nt) in the H3A helix of ITS2. In contrast, the ITS2 sequence of the ex-type strain of *C. eucalypti* (CBS 124764) possesses a hairpin loop in H4 that is longer by one nucleotide than that of all other strains in the clade. The grouping of strains in two Z clades is also supported by morphological differences. The ex-type strain of *C. guyanensis* [13] lacks intercalary phialides but forms prominent lateral phialides that are often aggregated in large groups (Fig. 16C–F), while *C. eucalypti* [91] is distinguished by the occasional presence of intercalary or short phialides without septa (Fig. 16H, I, M, N).

None of the differences recorded in the 2D structure of ITS1 and in the conserved part of ITS2 warrant recognition of two biological species according to the CBC species concept sensu Coleman [31] and the presence of two hCBCs between the ex-type strains of this clade indicate that they can theoretically interbreed. Whether these differences correspond with the process of speciation or adaptation to a new niche should be further investigated based on the examination of additional strains exhibiting the morphological characteristics of the ex-type strain of *C. eucalypti*. The strain MUCL 43737 is distant from other members of this clade in the four-gene ML analysis (Fig. 3) but in the BI analysis the branches of the clade (1.0) collapse. Because the main difference between the two species lies in the occasional occurrence of intercalary phialides in *C. eucalypti* (the shape and size of phialides and conidia of all isolates in this clade are comparable), we accept *C. guyanensis* as a name for the whole clade. The morphological variability among strains is noted in the key.

## Conclusions

Two novel evolutionary lineages were revealed in the Chaetothyriales based on the analysis of molecular, morphological and ITS structural data. The Cyphellophoraceae, introduced in our study, was consistently resolved as a robust clade based on analyses of ITS, other two ribosomal and three protein-coding gene sequences. The family accommodates dematiaceous microscopic fungi that are known to reproduce asexually only and includes medically important and saprobic fungi occupying a wide range of substrates. We emend *Cyphellophora* to include species with septate and nonseptate conidia formed on phialidic loci on phialides that can be intercalary, lateral or terminal and propose six new combinations in the genus. A second novel lineage was revealed within the Chaetothyriaceae, and is recognized here as the *Vonarxia*-group, it includes two *Cyphellophora* (*C. eugeniae* and *C. hylomeconis*), *Exophiala eucalyptorum* and *Vonarxia vagans*. Based on their distinctive morphologies and molecular data, the former *Cyphellophora* are introduced as type species of *Aphanophora* and *Camptophora*. *Capronia peltigerae,* known from lichen thalli and recently excluded from the Herpotrichiellaceae, was positioned with rock-inhabiting strains and other species currently referred to *Knufia*. We propose a new combination for this taxon in *Knufia*.

Phylogenetic results were supported by analyses of the secondary structure of the ITS1 and ITS2, and the identification of two evolutionary motifs (M2, M3) in the helix H3 of ITS2 molecule that characterize clades recognized as families. We also detected several double-sided (CBCs) and one-sided (hCBCs) substitutions in ITS1 and in the conserved regions of helices H2 and H3 of ITS2 that uniquely characterize the Cyphellophoraceae and taxa included in the *Vonarxia*-group, and which represent non-homoplasious synapomorphies in the Chaetothyriales.

The molecular and structural RNA data were combined with morphological and cultural characteristics to delimit the novel taxonomic groups. The morphology-based key is provided to facilitate identification of microscopic fungi accepted in the Cyphellophoraceae.

## Supporting Information

Table S1 **A list of fungi, isolate information and new sequences determined for this study and those retrieved from GeneBank.**
(DOC)
