# Correction: Novel Evolutionary Lineages Revealed in the Chaetothyriales (Fungi) Based on Multigene Phylogenetic Analyses and Comparison of ITS Secondary Structure

**DOI:** 10.1371/annotation/acfe0686-dd9d-4673-8cc9-3bb9f457a8c4

**Published:** 2013-11-19

**Authors:** Martina Réblová, Wendy A. Untereiner, Kamila Réblová

Under the section "Cyphellophora oxyspora" in the Results section, the species *Phialophora olivacea* is incorrectly referenced. The correct species is *Phialophora oxyspora.*

