# Supplementary material for: Novel Evolutionary Lineages Revealed in the Chaetothyriales (Fungi) Based on Multigene Phylogenetic Analyses and Comparison of ITS Secondary Structure
Source: PLoS One. 2013 May 28;8(5):e63547. doi: 10.1371/journal.pone.0063547 (PMC3665825; doi:10.1371/journal.pone.0063547)
Supplement: Table S1 — A list of fungi, isolate information and new sequences determined for this study and those retrieved from GeneBank. (DOC) [file pone.0063547.s001.doc]

**Table S1.** A list of fungi, isolate information and new sequences determined for this study and those retrieved from GeneBank.

| **Taxon** | **Source** | **Type** | **Substrate and locality** | **ITS** | **nuc28S** | **nuc18S** | ***mcm7*** | **β-tubulin** | ***rpb2*** |
| --- | --- | --- | --- | --- | --- | --- | --- | --- | --- |
| ***Aphanophora eugeniae*** | CBS 124105 | T | leaves of *Eugenia uniflora*, Brazil | FJ839617 | FJ839652 | - | **KC455266** | **KC455221** | - |
| ***Brycekendrickomyces acaciae*** | CBS 124104 | T | living leaves of *Acacia auriculiformis*, Indonesia | FJ839606 | FJ839641 | - | - | - | - |
| ***Camptophora hylomeconis*** | CBS 113311 | T | decaying leaves of *Hylomecon vernalis*, Korea | **KC455241** | EU035415 | **KC455295** | **KC455267** | **KC455222** | **KC455285** |
| ***Capronia pilosella*** | DAOM 216837 |  | on *Hypoxylon* sp., on *Fagus* sp., USA | - | DQ823099 | DQ823106 | - | - | - |
| ***Capronia semiimmersa*** | UAMH 10872 |  | decaying wood, Czech Republic | EU514691 | EU514691 | - | - | EU514704 | - |
| ***Ceramothyrium carniolicum*** | CBS 175.95 | T | leaves of *Pyrola rotundifolia*, Sweden | **KC455237** | **KC455251** | **KC455294** | **KC455265** | - | EF413630 |
| ***Ceramothyrium linnaeae*** | UPSC 2646 |  | leaves of *Linnaea borealis*, Sweden | - | - | AF022715 | - | - | AY485617 |
| ***Ceramothyrium thailandicum*** | MFLUCC 10-0079 |  | living leaves of *Lagerstroemia* sp., Thailand | HQ895838 | HQ895835 | - | - | - | - |
| ***Chaetothyrium brischofiacola*** | MFLUCC 10-0083 | T | living leaf of *Brischofia javanica*, Thailand | HQ895839 | HQ895836 | - | - | - | - |
| ***Cladophialophora carrionii*** | CBS 160.54 | T | man skin, Australia | AF050262 | FJ358234 | FJ358302 | - | - | - |
| ***Cladophialophora proteae*** | CBS 111667 | T | leaves of *Protea cynaroides*, South Africa | EU035411 | EU035411 | - | - | - | - |
| ***Cyphellophora ambigua*** | CBS 235.93 | T | man, toenail, Netherlands | JQ766431 | JQ766480 | - | - | JQ766340 | - |
| ***Cyphellophora europaea*** | CBS 101466 | T | man skin, foot, Netherlands | **KC455246** | **KC455259** | **KC455303** | **KC455276** | **KC455229** | - |
| ***Cyphellophora europaea*** | CBS 129.96 |  | man skin, toe, Germany | EF551553 | FJ358248 | FJ358317 | - | - | - |
| ***Cyphellophora europaea*** | CBS 218.78 |  | man, nail, Netherlands | JQ766441 | JQ766488 | - | - | JQ766366 | - |
| ***Cyphellophora guyanensis*** | MUCL 43737 | T | tropical leaf litter, French Guyana | **KC455240** | **KC455253** | **KC455296** | **KC455268** | **KC455223** | - |
| ***Cyphellophora guyanensis*** | CBS 126020 |  | rotting wood, Brazil | JQ766435 | JQ766484 | - | - | JQ766341 | - |
| ***Cyphellophora guyanensis*** | CBS 126014 |  | tucum palm, Brazil | JQ766434 | JQ766483 | - | - | JQ766339 | - |
| ***Cyphellophora guyanensis*** | CBS 125756 |  | roof of patient´s house, Brazil | JQ766433 | JQ766482 | - | - | JQ766338 | - |
| ***Cyphellophora guyanensis*** | CBS 124764 | T | leaves of *Eucalyptus* sp., Australia (*C. eucalypti*) | **KC455238** | **KC455254** | **KC455297** | **KC455269** | JQ766337 | **-** |
| ***Cyphellophora fusarioides*** | MUCL 44033 | T | man, bronchial lavage fluid, Israel | **KC455239** | **KC455252** | **KC455298** | **KC455270** | **KC455224** | - |
| ***Cyphellophora laciniata*** | CBS 190.61 | T | man skin, Switzerland | EU035416 | FJ358239 | FJ358307 | **KC455271** | JQ766329 | **KC455286** |
| ***Cyphellophora laciniata*** | CBS 124187 |  | bathroom, Netherlands | GQ426974 | - | - | - | - | - |
| ***Cyphellophora laciniata*** | MUCL 4293 |  | wood, Canada | GU225944 | - | - | - | - | - |
| ***Cyphellophora olivacea*** | CBS 122.74 | T | moist wall paper, Germany | **KC455247** | **KC455260** | - | **KC455277** | **KC455230** | - |
| ***Cyphellophora olivacea*** | CBS 123.74 |  | stem of *Chamaerops humili*, Netherlands | **KC455248** | **KC455261** | **KC455304** | **KC455278** | **KC455231** | - |
| ***Cyphellophora oxyspora*** | CBS 698.73 | T | decaying leaf of *Clerodendron monahassa*, Sri Lanka | **KC455249** | **KC455262** | **KC455305** | **KC455279** | **KC455232** | - |
| ***Cyphellophora oxyspora*** | IFM 51368 |  | data unpublished | AB190870 | - | - | - | - | - |
| ***Cyphellophora oxyspora*** | CBS 416.89 |  | man skin, Denmark | JQ766449 | JQ766497 | - | - | JQ766374 | - |
| ***Cyphellophora pauciseptata*** | CBS 284.85 | T | man skin, Netherlands | JQ766466 | JQ766515 | - | - | JQ766358 | - |
| ***Cyphellophora pluriseptata*** | CBS 286.85 | T | man skin, Netherlands | **KC455242** | **KC455255** | **KC455299** | **KC455272** | **KC455225** | **KC455287** |
| ***Cyphellophora reptans*** | CBS 113.85 | T | food, Sweden | EU514699 | EU514699 | **KC455306** | **KC455280** | **KC455233** | - |
| ***Cyphellophora reptans*** | CBS 458.92 |  | man skin, Netherlands | **KC455250** | **KC455263** | **KC455307** | **KC455281** | JQ766372 | - |
| ***Cyphellophora reptans*** | CBS 120903 |  | drinking water, Germany | JQ766448 | JQ766496 | - | - | JQ766373 | - |
| ***Cyphellophora reptans*** | CBS 152.90 |  | man, nail, Netherlands | JQ766446 | JQ766494 | - | - | JQ766371 | - |
| ***Cyphellophora sessilis*** | CBS 243.85 | T | resin of *Picea abies, Netherlands* | EU514700 | EU514700 | **KC455308** | **KC455282** | **KC455234** | **KC455288** |
| ***Cyphellophora sessilis*** | CBS 238.93 |  | styrene, Netherlands | AY857541 | **KC455264** | **KC455309** | **KC455283** | **KC455235** | **KC455289** |
| ***Cyphellophora sessilis*** | ZJ81-D7 |  | bamboo, China | GU981735 | - | - | - | - | - |
| ***Cyphellophora suttonii*** | CBS 449.91 | T | subcutaneous lesion in dog´s ear, USA | **KC455243** | **KC455256** | **KC455300** | **KC455273** | **KC455226** | **KC455290** |
| ***Cyphellophora vermispora*** | CBS 228.86 | T | root of *Triticum aestivum*, Germany | **KC455244** | **KC455257** | **KC455301** | **KC455274** | **KC455227** | **KC455291** |
| ***Cyphellophora vermispora*** | CBS 227.86 |  | root of *Hordeum vulgare*, Germany | JQ766425 | JQ766474 | - | - | JQ766331 | - |
| ***Cyphellophora vermispora*** | CBS 122852 |  | man skin, foot, Netherlands | JQ766427 | JQ766476 | - | - | JQ766333 | - |
| ***Exophiala dermatitidis*** | CBS 207.35 | T | man, locality unknown | - | DQ823100 | DQ823107 | - | - | - |
| ***Exophiala eucalyptorum*** | CBS 121638 | T | leaf litter of *Eucalyptus* sp., New Zealand | **KC455245** | **KC455258** | **KC455302** | **KC455275** | **KC455228** | **KC455292** |
| ***Exophiala jeanselmei*** | CBS 507.90 |  | man, Uruguay | - | FJ358242 | FJ358310 | - | - | - |
| ***Exophiala pisciphila*** | CBS 537.73 | T | *Ictalurus punctatus*, USA | - | DQ823101 | DQ823108 | - | - | - |
| ***Glyphium elatum*** | CBS 268.34 |  | *Salix*, USA |  | AF346420 | AF346419 |  |  |  |
| ***Knufia cryptophialidica*** | DAOM 216555 | T | black gall of *Populus tremuloides*, Canada | JN040501 | JN040501 | EF137364 | - | - | - |
| ***Knufia cryptophialidica*** | DAOM 216552 |  | black gall of *Populus tremuloides*, Canada | JN040502 | - | - | - | - |  |
| ***Knufia endospora*** | DAOM 241199 |  | Twig of *Populus* *balsamifera*, Canada | JN040511 | - | - | - | - | - |
| ***Knufia endospora*** | UAMH 10396 | T | twig of *Populus tremuloides*, Canada | - | - | JN040509 | - | - | - |
| ***Knufia epidermidis*** | CBS 120353 | T | foot infection, China | EU730589 | FJ355954 | FJ355953 | - | - | - |
| ***Knufia chersonesos*** | IMI 389175 | T | marble, Ukraine | AJ507323 | AJ507323 | - | - | - | - |
| ***Knufia chersonesos*** | CBS 726.95 | T | marble, Sicily (*Sarcinomyces petricola*) | AJ244275 | - | - | - | - | - |
| ***Knufia perforans*** | CBS 885.95 | T | marble, Greece | AJ244230 | FJ358237 | EF137365 | - | - | - |
| ***Knufia peltigerae*** | UAMH 11090 |  | thalli of *Peltigera rufescens*, Luxembourg | HQ709322 | HQ613813 | HQ613815 | - | - | - |
| ***Metulocladosporiella musae*** | CBS 161.74 | T | *Musa*, Honduras | AY186199 | DQ008161 | - | - | - | - |
| ***Metulocladosporiella musicola*** | CBS 110960 | T | *Musa acuminata*, South Africa | DQ008127 | DQ008159 | - | - | - | - |
| ***Phaeococcomyces catenatus*** | CBS 650.76 | T | air isolate, Switzerland | AF050277 | AF050277 | FJ358316 | - | - | - |
| ***Phaeococcomyces catenatus*** | CPC 13707 | T | leaves of *Eucalyptus placita*, Australia (*E. placitae*) | EU040215 | EU040215 | - | - | - | - |
| ***Phaeosaccardinula ficus*** | MFLUCC 10-0080 |  | living leaf of *Ficus* sp., Thailand | HQ895840 | HQ895837 | - | - | - | - |
| ***Phialophora verrucosa*** | MUCL 9760 |  | man, Brazil | AF050281 | EF413615 | EF413614 | - | EU514714 | - |
| ***Ramichloridium anceps*** | CBS 181.65 |  | soil under Thuja plicata, Canada | - | DQ823102 | AY554292 | - | - | - |
| ***Trichomerium deniqulatum*** | MFLUCC 10-0884 | T | living leaf of *Psidium guajava*, Thailand | JX313654 | JX313660 | - | - | - | - |
| ***Trichomerium foliicola*** | MFLUCC 10-0054 |  | living leaf of *Mangifera indica*, Thailand | JX313651 | JX313657 | - | - | - | - |
| ***Trichomerium foliicola*** | MFLUCC 10-0058 |  | living leaf of *Phoenix dactylifera*, Thailand | JX313653 | JX313659 | - | - | - | - |
| ***Trichomerium foliicola*** | MFLUCC 10-0078 | T | living leaf of *Murraya paniculata*, Thailand | JX313655 | JX313661 | - | - | - | - |
| ***Trichomerium foliicola*** | MFLUCC 10-0073 |  | living leaf of *Psidium guajava*, Thailand | JX313652 | - | - | - | - | - |
| ***Trichomerium gleosporum*** | MFLUCC 10-0087 |  | living leaf of *Ficus* sp., Thailand | JX313656 | JX313662 | - | - | - | - |
| ***Vonarxia vagans*** | CBS 123533 | T | living leaves of *Stenocalyx uniflorus*, Brazil | FJ839636 | FJ839672 | **KC455310** | **KC455284** | **KC455236** | **KC455293** |
| ***Vonarxia vagans*** | CPC 15152 |  | living leaves of *Stenocalyx uniflorus*, Brazil | FJ839637 | FJ839673 | - | - | - | - |

GenBank accession numbers in bold were generated for this study.
